# Supplementary material for: Intraoperative cell-salvaged versus allogeneic red blood cell transfusions in high-bleeding-risk cardiovascular surgery: Protocol for a single-center, randomized, parallel-group, noninferiority trial
Source: PLoS One. 2025 Oct 24;20(10):e0334397. doi: 10.1371/journal.pone.0334397 (PMC12551814; doi:10.1371/journal.pone.0334397)
Supplement: S2 File — (DOCX) [file pone.0334397.s002.docx]

Intraoperative cell-salvaged versus allogeneic red blood cell transfusions in high-bleeding-risk cardiovascular surgery: Protocol for a single-center, randomized, parallel-group, noninferiority trial

**S**tudy on the **E**ffect of intraoperative cell-salvaged versus allogeneic transfusion on postoperative blood **L**oss and coagulation **F**unction: SELF Trial

Principal Investigator: Akito Tsukinaga, Department of Anesthesiology

Version 1.1: July 11, 2024

Version 1.2: September 13, 2024

Version 1.3: December 4, 2024

Version 1.4: August 12, 2025

Version 1.5: October 1, 2025

| **Compliance with the Approved Study Protocol Reviewed by the Ethics Committee**  **This study will be conducted in accordance with the ethical principles of the Declaration of Helsinki and the "Ethical Guidelines for Life Science and Medical Research Involving Human Subjects." The study will respect the fundamental human rights of the subjects and adhere to the study protocol approved by the Ethics Committee and authorized by the head of the research institution.** |
| --- |

# **0. Study Overview**

| Study Title | | Intraoperative cell-salvaged versus allogeneic red blood cell transfusions in high-bleeding-risk cardiovascular surgery: Protocol for a single-center, randomized, parallel-group, noninferiority trial |
| --- | --- | --- |
| Principal Investigator | | Akito Tsukinaga |
| Study Objective | | The objective of this study is to evaluate, through a single-blind randomized controlled non-inferiority trial, the hypothesis that transfusing intraoperative cell salvage blood for anemia after CPB in high-risk bleeding cardiovascular surgery does not result in greater blood loss in the ICU compared to transfusing only allogeneic RBC, without using cell salvage blood. |
| Study Design | | Single-blind, randomized, parallel-group, non-inferiority trial |
| Study Participants | | Patients undergoing cardiovascular surgery with CPB |
| Eligibility Criteria | Inclusion Criteria | <Provisional registration>   1. Patients who have given informed consent. 2. Patients aged 40 years or older at the time of consent acquisition. 3. Patients scheduled to undergo elective cardiovascular surgery using CPB with a median sternotomy approach. 4. Patients meeting any of the following conditions (high bleeding risk cases):   (a) Re-operative chest surgery following a prior median sternotomy approach.  (b) Patients undergoing surgery involving the aortic root (Ross procedure, aortic valve replacement with annular enlargement, aortic root replacement), ascending aorta replacement, or arch aorta replacement.  (c) Patients undergoing surgery for two or more valve diseases, excluding tricuspid annuloplasty.  (d) Patients undergoing CABG combined with valve surgery, excluding tricuspid annuloplasty.  <Final registration>  Patients who have received allogeneic blood transfusion during CPB. |
|  | Exclusion Criteria | <Provisional registration>   1. Patients scheduled for descending aorta replacement, thoracoabdominal aorta replacement, heart transplantation, ventricular assist device implantation, or pulmonary valve replacement. 2. Patients with an extremely high risk of bleeding. 3. Patients who have not adhered to the prescribed withdrawal periods for antiplatelet or anticoagulant medications. 4. Patients with active bacterial or viral infections. 5. Pregnant or breastfeeding patients. 6. RhD negative patients. 7. Patients with positive irregular antibodies. 8. Other patients deemed unsuitable for participation by the principal investigator or study team.   <Final registration>   1. Patients who fail to adhere to the prescribed withdrawal periods for antiplatelet or anticoagulant medications after pre-registration and before the surgery date. 2. If the surgical procedure is changed between pre-registration and the surgery date, or during the surgery, and it conflicts with the pre-registration inclusion criteria or meets any of the pre-registration exclusion criteria. |
| Investigational Product | Intervention group | Intraoperative cell salvage blood will be used preferentially over allogeneic RBC products for anemia after CPB. If anemia is not corrected by cell salvage blood, or if it is anticipated to remain uncorrected, allogeneic RBC will be transfused. |
|  | Control group | Allogeneic RBC will be transfused for anemia after CPB, without the use of intraoperative cell salvage. |
| Overview of Study Procedure | | Informed consent and pre-registration will be obtained prior to surgery, and post-registration will occur during the surgery. After registration, participants will be randomly assigned in a 1:1 ratio to either the cell salvage group or the allogeneic RBC transfusion group. The randomization will use stratified block randomization. Throughout the study period, blinding will be maintained for the participants, evaluators, surgeons, surgical assistants, and the physicians involved in postoperative management. The investigational product will be administered when anemia with a hemoglobin level below 10.0g/dL is confirmed or anticipated based on laboratory tests. |
| Duration of Intervention | | The investigational product will be administered from the time of CPB weaning until the patient exits the operating room. |
| Outcome | Primary outcome | Total drainage volume within 12 hours of ICU admission |
|  | Secondary outcome | 1. The volume of allogeneic RBC transfused from the start of general anesthesia to 12 hours after ICU admission 2. The incidence of re-sternotomy or re-operation for blood clot removal within 48 hours of ICU admission 3. The proportion of patients with total drainage volume exceeding 1000mL within 12 hours of ICU admission |
|  | Safety outcome | 1. Prevalence of any of the following infections: surgical site infection, mediastinitis, urinary tract infection, respiratory infection, central nervous system infection, gastrointestinal system infection, skin and soft tissue infection, or other infection 2. Prevalence of gross hematuria upon ICU admission 3. Prevalence of acute kidney injury within 7 days after surgery, defined in accordance with the Kidney Disease: Improving Global Outcomes criteria. |
| Planned Sample Size | | 142 cases (71 in the intraoperative cell salvage group and 71 in the allogeneic RBC group). |
| Rationale for Sample Size | | Based on preliminary data from patients meeting the eligibility criteria, the average drainage volume within 12 hours of ICU admission for 59 cases using only allogeneic RBC was 674 mL with a standard deviation of 400 mL. Using a non-inferiority margin of 200 mL and power calculations (α = 0.025, β = 0.2), the required sample size was calculated as 128 (64 per group). Accounting for a 10% dropout rate, the final sample size was set at 142 (71 per group). |
| Planned Study Period | | Study Duration: Research approval date – March 31, 2030Enrollment Period: Research approval date – September 30, 2026Follow-up Period: Research approval date – November 4, 2026 |
| Study Secretariat | | National Cerebral and Cardiovascular Center, Department of Transfusion Address: 6-1 Kishibe Shinmachi, Suita City, Osaka 564-8565, JapanTEL: +81-6-6170-1069 (Extension 40563)FAX: +81-6-6170-1953e-mail：onishi.yuko@ncvc.go.jp |

Patients scheduled for elective cardiovascular surgery using CPB with high bleeding risk

Informed Consent

Eligibility Confirmation and provisional registration

Allogeneic RBC Transfusion during CPB

Not Transfused

Transfused

Not registerd

Final registration and randomization

Salvaged RBC group

Administration of salvaged RBC prioritized over allogeneic RBC from weaning of CPB until the patient exits the operating room

　Allogeneic blood group

Only allogeneic RBC transfusion from weaning of CPB until the patient exits the the operating room

**Observation Period**: **30days**

The primary outcome will be the measurement of the total drainage volume within 12 hours of ICU admission. As a secondary outcome, patients will be followed for 30 days after the administration of the investigational treatment.

Abbreviations

| AE | Adverse event |
| --- | --- |
| ACT | Activated clotting time |
| ALT | Alanine aminotransferase |
| APTT | Activated partial thromboplastin time |
| AST | Aspartate aminotransferase |
| ATIII | Anti thrombin III |
| BNP | Brain natriuretic peptide |
| CABG | Coronary artery bypass grafting |
| CDC | Centers for disease control and prevention |
| CK | Creatine kinase |
| CPB | Cardiopulmonary bypas |
| CRF | Case report forms |
| CRP | C-reactive protein |
| FAS | Full analysis set |
| FBS | Fast blood sugar |
| FDP | Fibrin/fibrinogen degradation products |
| FFP | Fresh frozen plasma |
| ICU | Intensive care unit |
| ITT | Intent-to-treat population |
| LDH | Lactate dehydrogenase |
| PPS | Per protocol set |
| PT-INR | Prothrombin time-international normalized ratio |
| RBC | Red blood cell |
| SAE | Serious adverse event |
| SSI | Surgical site infection |

**Table of contents**

[**0. Study Overview** i](#_Toc193809699)

[**1.** **Background and rationale** 1](#_Toc193809700)

[**1.1.** **Current Status of the Target Disease in Japan and Abroad** 1](#_Toc193809701)

[**1.2.** **History and Overview of Standard Treatments** 1](#_Toc193809702)

[**1.3.** **Current Standard Treatments and Outcomes** 1](#_Toc193809703)

[**1.4.** **Challenges and Uncertainties in the Current Standard Treatment Leading to the Necessity of This Clinical Trial** 1](#_Toc193809704)

[**1.5.** **Validity of the Trial Implementation.** 2](#_Toc193809705)

[**2.** **Objective** 2](#_Toc193809706)

[**3.** **Trial Design** 2](#_Toc193809707)

[**4.** **Outcomes** 3](#_Toc193809708)

[**4.1.** **Primary Outcome** 3](#_Toc193809709)

[**4.2.** **Secondary Outcomes** 3](#_Toc193809710)

[**4.3.** **Safety Outcomes** 3](#_Toc193809711)

[**4.4.** **Experimental outcomes** 3](#_Toc193809712)

[**5.** **Investigational Product Information** 4](#_Toc193809713)

[**5.1.** **Investigational Product** 4](#_Toc193809714)

[**5.1.1.** **Name, Content, etc.** 4](#_Toc193809715)

[**5.1.2.** **Packaging and Labeling** 4](#_Toc193809716)

[**5.1.3.** **Quality Control** 4](#_Toc193809717)

[**5.1.4.** **Flow from Blood Collection to Transfusion** 4](#_Toc193809718)

[**5.1.5.** **Settings for Cell Saver Elite** 4](#_Toc193809719)

[**5.2.** **Control Product** 5](#_Toc193809720)

[**5.2.1.** **Name, Content, etc.** 5](#_Toc193809721)

[**5.2.2.** **Packaging and Labeling** 5](#_Toc193809722)

[**5.2.3.** **Quality Control** 5](#_Toc193809723)

[**6.** **Participants** 5](#_Toc193809724)

[**6.1.** **Imnclusion and Exclusion Criteria for Provisional Registration** 5](#_Toc193809725)

[**6.1.1.** **Inclusion Criteria** 5](#_Toc193809726)

[**6.1.2.** **Exclusion Criteria** 6](#_Toc193809727)

[**6.1.3.** **Rationale for Criteria** 7](#_Toc193809728)

[**6.1.3.1.** **Rationale for Inclusion Criteria** 7](#_Toc193809729)

[**6.1.3.2.** **Rationale for Exclusion Criteria** 7](#_Toc193809730)

[**6.2.** **Selection and Exclusion Criteria for Registration** 7](#_Toc193809731)

[**6.2.1.** **Criterias of choice** 7](#_Toc193809732)

[**6.2.2.** **Exclusion Criteria** 7](#_Toc193809733)

[**6.2.3.** **Rationale for Criteria** 7](#_Toc193809734)

[**6.2.3.1.** **Rationale for Inclusion Criteria** 7](#_Toc193809735)

[**6.2.3.2.** **Rationale for Exclusion Criteria** 7](#_Toc193809736)

[**7.** **Study Procedures** 7](#_Toc193809737)

[**7.1.** **Informed Consent** 7](#_Toc193809738)

[**7.1.1.** **Voluntary Participation** 7](#_Toc193809739)

[**7.1.2.** **Procedure for Obtaining Informed Consent** 8](#_Toc193809740)

[**7.1.3.** **Re-Consent** 8](#_Toc193809741)

[**7.1.4.** **Withdrawal of Consent** 8](#_Toc193809742)

[**7.2.** **Provisional Registration** 8](#_Toc193809743)

[**7.3.** **Final Registration** 8](#_Toc193809744)

[**7.4.** **Randomization and Blinding** 9](#_Toc193809745)

[**7.5.** **Administration of the Study Treatment** 9](#_Toc193809746)

[**7.5.1.** **Salvaged RBC Group (Investigational Product Group)** 9](#_Toc193809747)

[**7.5.2.** **Allogeneic RBC Group (Control Product Group)** 9](#_Toc193809748)

[**7.5.3.** **Criteria for Discontinuation of Study Treatment** 10](#_Toc193809749)

[**7.6.** **Pre- and Concomintant Treatments** 10](#_Toc193809750)

[**7.6.1.** **Pre-Treatment Defined by the Study** 10](#_Toc193809751)

[**7.6.2.** **Concomintant Therapies** 10](#_Toc193809752)

[**7.6.3.** **Post-Treatment** 10](#_Toc193809753)

[**7.7.** **Discontinuation Criteria and Procedures for Study Treatment** 11](#_Toc193809754)

[**7.7.1.** **Procedures for Discontinuation of Study Treatment** 11](#_Toc193809755)

[**7.8.** **Discontinuation Criteria and Procedures for Study Participation** 11](#_Toc193809756)

[**7.8.1.** **Discontinuation Criteria** 11](#_Toc193809757)

[**7.8.2.** **Procedures for Discontinuation of Participation** 11](#_Toc193809758)

[**8.** **Observation and Examination Items and Schedule** 12](#_Toc193809759)

[**8.1.** **Visit 1. Consent Acquisition, Eligibility Confirmation, and Provisional Registration** 12](#_Toc193809760)

[**8.2.** **Visit 2. Final Registration and Randomization (Baseline)** 12](#_Toc193809761)

[**8.3.** **Visit 3. During Study Treatment** 13](#_Toc193809762)

[**8.4.** **Visit 4. End of Study** 14](#_Toc193809763)

[**8.5.** **When the study is stopped early (date of decision to discontinue+3)** 14](#_Toc193809764)

[**8.6.** **Definition of observation and inspection items** 17](#_Toc193809765)

[**8.6.1.** **Definition of the disease** 17](#_Toc193809766)

[**9.** **Evaluation and Reporting of AEs** 17](#_Toc193809767)

[**9.1.** **Definition of AEs** 17](#_Toc193809768)

[**9.2.** **Response to the Occurrence of AEs** 17](#_Toc193809769)

[**9.3.** **Evaluation of AEs** 17](#_Toc193809770)

[**9.3.1.** **Definition of SAEs** 17](#_Toc193809771)

[**9.3.2.** **Severity of AEs** 18](#_Toc193809772)

[**9.3.3.** **Causal Relationship with the Study Product** 18](#_Toc193809773)

[**9.4.** **Reporting of AEs** 18](#_Toc193809774)

[**9.4.1.** **Reporting Period for AEs** 18](#_Toc193809775)

[**9.4.2.** **Reporting Procedure for SAEs** 18](#_Toc193809776)

[**9.5.** **Anticipated AEs** 19](#_Toc193809777)

[**9.5.1.** **Common AEs** 19](#_Toc193809778)

[**9.5.2.** **Specific to Allogeneic RBC Transfusion** 19](#_Toc193809779)

[**9.5.3.** **Specific to Salvaged RBC Transfusion** 19](#_Toc193809780)

[**10.** **Study Period** 20](#_Toc193809781)

[**11.** **Target Sample Size** 20](#_Toc193809782)

[**12.** **Statistics Considerations** 20](#_Toc193809783)

[**13.** **Ethical Considerations** 20](#_Toc193809784)

[**13.1.** **Applicable Guidelines and Regulations** 20](#_Toc193809785)

[**13.2.** **Procedures** **for Study Implementation** 20](#_Toc193809786)

[**13.3.** **Reporting to the Head of the Institution and Ethics Committee** 20](#_Toc193809787)

[**13.4.** **Overall Assessment of Benefits and Burdens to Study Participants** 21](#_Toc193809788)

[**13.4.1.** **Benefits to participants and potential benefits of the study** 21](#_Toc193809789)

[**13.4.2.** **Burden on study participants** 21](#_Toc193809790)

[**13.4.3.** **Anticipated risks to study participants** 21](#_Toc193809791)

[**13.4.4.** **Measures to minimize risk** 21](#_Toc193809792)

[**13.4.5.** **Overall Evaluation** 21](#_Toc193809793)

[**13.4.6.** **Compensation for participation** 21](#_Toc193809794)

[**13.5.** **Handling of inquiries from participants and their representatives** 21](#_Toc193809795)

[**13.6.** **Preparation and Revision of the Study Information Sheet and Consent Form** 22](#_Toc193809796)

[**13.6.1.** **Preparation of documents and approval by the institution** 22](#_Toc193809797)

[**13.6.2.** **Required content of the study information sheet** 22](#_Toc193809798)

[**13.6.3.** **Revision of the study information sheet and consent form** 23](#_Toc193809799)

[**14.** **Protection of Personal Information** 23](#_Toc193809800)

[**15.** **Data Management** 23](#_Toc193809801)

[**15.1.** **Preparation of CRF** 23](#_Toc193809802)

[**15.2.** **Amendments or Corrections to the CRFs** 24](#_Toc193809803)

[**15.3.** **Attribution of collected data** 24](#_Toc193809804)

[**16.** **Storage of Samples and Data** 24](#_Toc193809805)

[**16.1.** **Method of Storage** 24](#_Toc193809806)

[**16.2.** **Transfer of Samples/Data to External Organizations** 24](#_Toc193809807)

[**16.3.** **Retention Period of samples and information** 24](#_Toc193809808)

[**16.4.** **Future Use of Data** 24](#_Toc193809809)

[**17.** **Quality Control & Quality Assurance** 25](#_Toc193809810)

[**17.1.** **Compliance, Deviations, and Amendments** 25](#_Toc193809811)

[**17.2.** **Protocol Amendments** 25](#_Toc193809812)

[**17.3.** **Monitoring** 25](#_Toc193809813)

[**17.4.** **Audit** 25](#_Toc193809814)

[**18.** **Termination or Discontinuation of the Study** 25](#_Toc193809815)

[**18.1.** **Study Termination** 25](#_Toc193809816)

[**18.2.** **Discontinuation or Suspension of the Study** 25](#_Toc193809817)

[**18.2.1.** **Criteria for Discontinuation or Suspension** 25](#_Toc193809818)

[**18.2.2.** **Procedures for Discontinuation or Suspension** 26](#_Toc193809819)

[**19.** **Funding and Conflict of Interest** 26](#_Toc193809820)

[**19.1.** **Funding** 26](#_Toc193809821)

[**19.2.** **Conflict of Interest** 26](#_Toc193809822)

[**20.** **Compensation for Study-related Injury** 26](#_Toc193809823)

[**21.** **Post-study Medical Care for Participants** 27](#_Toc193809824)

[**22.** **Handling of Study Results and Health Information** 27](#_Toc193809825)

[**23.** **Publication and Ownership of Study Results** 27](#_Toc193809826)

[**23.1.** **Study Registration** 27](#_Toc193809827)

[**23.2.** **Publication of Study Results** 27](#_Toc193809828)

[**23.3.** **Ownership of Study Data and Results** 27](#_Toc193809829)

[**24.** **Study Implementation Structure** 27](#_Toc193809830)

[**25.** **Oversight of Contracted Organizationss** 27](#_Toc193809831)

[**26.** **References** 27](#_Toc193809832)

# **Background and rationale**

## **Current Status of the Target Disease in Japan and Abroad**

In Japan, an aging population and declining birthrate are expected to result in decreased blood donations and an increased demand for blood products. It is estimated that there will be a shortfall of approximately 330,000–650,000 blood donations by 2025. [1] Therefore, reducing the use of blood products is an urgent issue.

Over 70,000 cardiovascular surgeries are performed annually in Japan. [2] Among medical specialties, cardiovascular surgery ranks second after hematology in the use of RBC and platelets and first in the use of FFP. [3] Reducing blood transfusion volumes in major cardiovascular surgeries is a major challenge.

## **History and Overview of Standard Treatments**

The standard method for rapidly correcting anemia during major cardiovascular surgery is allogeneic RBC transfusion.

## **Current Standard Treatments and Outcomes**

Although screening for known viruses has reduced the incidence of infection from allogeneic RBC transfusions, the risk of transmission of unknown viruses remains a concern. Furthermore, studies have reported that allogeneic RBC transfusions are associated with a higher risk of surgical site **infections**[4], **pneumonia**[5], and **renal dysfunction**[6] after major cardiovascular surgeries.

In CABG, the use of allogeneic RBC is associated with an increased risk of mortality, with a reported risk ratio of 1.7 (95% confidence interval: 1.4–2.0).[7]

## **Challenges and Uncertainties in the Current Standard Treatment Leading to the Necessity of This Clinical Trial**

One approach to avoid the issues associated with allogeneic RBC transfusions is autologous blood transfusion. There are three methods of autologous blood transfusion: pre-donation (or blood banking), dilutional, and cell salvage transfusion. Among these, cell salvage is commonly used in major cardiovascular surgery.

In cell salvage, a specialized device is used to collect blood lost during surgery from the surgical field. The collected blood is passed through a filter in a reservoir to remove foreign matter and stored. Once a certain amount of blood has been collected, it is concentrated and washed, and then stored in a blood bag to be returned to the patient.

In heart and large vessel surgeries, where blood loss tends to be relatively high, blood recovery offers the advantage of reducing the need for allogeneic RBC transfusion, helping decrease the risks associated with donor blood transfusions.[8-11]。A systematic review and meta-analysis published by Cochrane in 2023 reported that the use of blood recovery during heart surgery with CPB resulted in a reduction of 1.47 units of allogeneic RBC transfusion (95% confidence interval: 0.36-2.59).[12] A facility in Japan, which performed approximately 1,100 cases of cardiac and vascular surgery over a 3-year period, reported that 78% of these cases involved blood recovery. As a result, a total of 9,301 units of allogeneic RBC transfusion were reduced over the 3 years.[13] Overseas guidelines recommend the use of blood recovery during cardiac surgery involving CPB.[14, 15]

However, there are reports that administering large amounts of salvaged RBC can cause coagulopathy due to residual heparin that was not completely removed during the washing process.[11] In a systematic review and meta-analysis by Cochrane, which reported a reduction in the use of allogeneic RBC transfusions with the use of salvaged RBC, the study population largely consisted of procedures with low blood loss risks, where transfusions were often not required. Therefore, the issue of residual heparin in salvaged RBC causing complications after the antagonism of heparin, particularly in high-risk surgical procedures that require relatively large amounts of transfusions, remains unresolved. Specifically, whether salvaged RBC can be used without increasing bleeding after separation from CPB remains unclear. According to a survey conducted among major facilities in Japan performing cardiovascular surgery, 40% of the cases had salvaged RBC prepared but discarded without being used. Therefore, it is urgent to to investigate the impact of salvaged RBC transfusions on blood loss in patients with a high risk of bleeding.

## **Validity of the Trial Implementation.**

If this study demonstrates that salvaged RBC transfusions do not increase blood loss compared with allogeneic RBC transfusions, it could lead to a reduction in the use of allogeneic RBC products and avoidance of adverse effects associated with them, thereby contributing to the maintenance of the blood supply system, medical economics, and ultimately patient benefit.

# **Objective**

This study aims to evaluate, through a single-blind randomized controlled non-inferiority trial, the hypothesis that intraoperative salvaged RBC transfusion for anemia after CPB in high-risk cardiovascular surgery does not result in greater postoperative bleeding compared to a transfusion strategy using only allogeneic RBC

# **Trial Design**

1. Design

- Type of control: Standard treatment control
- Design characteristics: Parallel group comparison
- Randomization: Yes (stratified block randomization)
- Blinding: Single-blind (participant blinded)
- Type of trial: Non-inferiority trial

1. Invasiveness: Yes (exceeds minimal invasiveness)
2. Intervention: Yes
3. Use of samples: New sample are collected
4. Use of information: New information is collected, and existing information is also used

# **Outcomes**

## **Primary Outcome**

Postoperative chest tube blood loss within 12 hours from the end of surgery. If re-thoracotomy for bleeding or hematoma removal is required within 12 hours, the intraoperative blood loss during re-thoracotomy is added to the total for calculation.

## **Secondary Outcomes**

1. Allogeneic RBC transfusion volume during surgery and for 12 hours from the end of surgery
2. Prevalence of re-thoracotomy within 48hours from the end of surgery
3. Prevalence of the postoperative chest tube blood loss within 12 hours from the end of surgery ≥1000mL

### **Safety Outcomes**

1. Prevalence of any of the following infections, defined according to the CDC diagnostic criteria[16]: surgical site infection, mediastinitis, urinary tract infection, respiratory infection, central nervous system infection, gastrointestinal infection, skin and soft tissue infection, etc.
2. Prevalence of gross hematuria upon ICU admission
3. Prevalence of acute kidney injury within 7 days after surgery, defined in accordance with the Kidney Disease: Improving Global Outcomes criteria.[17]

Safety outcomes 2) and 3) are set as indicators of mechanical hemolysis, one of the potential adverse events associated with salvaged RBC transfusion.

### **Experimental outcomes**

1. Results of blood viscoelastic testing, prothrombin time, activated partial thromboplastin time, and platelet count
2. Transfusion volume during surgery and for 12 hours from the end of surgery (FFP without cryoprecipitate/cryoprecipitate/platelet concentrate)
3. Duration of surgery (hours)
4. Duration of CPB (hours)
5. Duration of postoperative mechanical ventilation (hours)
6. Duration of ICU stay (hours)
7. Mortality (all-cause death and cardiovascular death)
8. Heparin concentration of salvaged RBC after processing remaining blood in the CPB circuit in the cell salvage device
9. Amount of protamine sulfate in the OR and within 12 hours from the end of surgery.

※Cardiovascular death is defined as death caused by the following factors:

1. Sudden cardiac death
2. Heart failure
3. Myocardial infarction
4. Stroke
5. Other cardiovascular-related causes (e.g., aneurysm, pulmonary embolism)

# **Investigational Product Information**

## **Investigational Product**

### **Name, Content, etc.**

Salvaged RBC transfusion（Cell Saver Elite, Hemonetics）

### **Packaging and Labeling**

The salvaged RBC is contained in a dedicated bag attached to the Cell Saver Elite device.

### **Quality Control**

The collected blood should be administered within 4 hours after recovery process is completed.

### **Flow from Blood Collection to Transfusion**

1. Following the instructions in the user manual, disposable components and other necessary parts are attached to the autotransfusion device.
2. The aspiration line bag is opened aseptically, and the sterilized circuit wrapped in sterilization paper is passed to the surgical field.
3. The aspiration line is received from the surgical field and aseptically connected to the reservoir.
4. The suction pressure is set. To prevent hemolysis, the suction pressure should be set to a maximum of -150 mmHg; however, in cases of rapid bleeding, the suction pressure may be temporarily increased to 250 mmHg.
5. The reservoir is primed with heparinized saline (15,000 units of heparin in 500 mL of saline solution).
6. The amount of heparinized saline dripping into the aspiration line is adjusted according to the amount of bleeding. Typically, 15 mL of heparinized saline is added to every 100 mL of collected blood.
7. Bleeding from the surgical field is aspirated using the Cell Saver Elite device. During CPB (after the ACT exceeds 200 s and before protamine is administered), aspiration should be performed inside the CPB circuit.
8. The collection, concentration, and washing procedures are performed according to the user manual.
9. After washing, the blood is returned to the dedicated bag and transfused to the patient using a blood transfusion filter.

### **Settings for Cell Saver Elite**

Bowl size: 225mL

Suction pressure: "Smart Suction" mode

Fat Removal: Off

Automatic washing: ON

Treat the blood before the enrichment of the bowl is completed: ask

Pump Flow Control: ON

Concentrator pump flow: 500 mL/h

Minimum cleaning volume: 1000 mL

Washing pump flow rate: 450mL/h

Smart Empty: ON

High-speed mode concentrator pump flow: 800 mL/h

High-speed mode washing pump flow rate: 800 mL/h

## **Control Product**

### **Name, Content, etc.**

Allogeneic RBC transfusion

### **Packaging and Labeling**

Contained in a bag visually distinct from the dedicated salvaged blood transfusion bag.

### **Quality Control**

The samples are stored under refrigeration.

# **Participants**

Eligibility is assessed in two stages: provisional and final registration. Participants who meet all the inclusion criteria and none of the exclusion criteria are considered eligible for each stage.

## **Imnclusion and Exclusion Criteria for Provisional Registration**

Patients who meet all the inclusion criteria and do not meet any of the exclusion criteria are provisionally enrolled.

## **Inclusion Criteria**

1. Patients with consent from the patient
2. Patients ≥ 40 years of age at the time of obtaining consent
3. Patients who use a heart-lung machine and undergo cardiovascular surgery using a median sternotomy approach on an elective basis (surgery application was made by 24 o'clock the day before the surgery date)
4. Patients who fall under any of the following (fall under the case of high risk of bleeding)
   1. Resumption thoracic surgery with a history of cardiac large vessel surgery with a median sternotomy approach in the past
   2. Patients undergoing aortic base surgery (Ross surgery, aortic valve replacement with annular enlargement, aortic base replacement), ascending aortic replacement, or arch aortic replacement
   3. Patients undergoing valvular disease surgery of 2 or more valves, except tricuspid annuloplasty
   4. Patients who undergo CABG combined with valvular disease surgery excluding tricuspid annuloplasty

## **Exclusion Criteria**

1. Patients undergoing descending aortic replacement, thoracoabdominal aortic replacement, heart transplantation, ventricular assist device transplantation, or pulmonary valve replacement
2. Extremely high risk of bleeding, defined as patients with three or more prior cardiovascular surgeries via median sternotomy. For other cases in which the bleeding risk is considered to be extremely high, the final decision on exclusion will be made through discussion between the surgeon and the anesthesiologist.
3. Patients who do not comply with the preoperative antiplatelet or anticoagulant withdrawal period (see Table 1)
4. Patients with active bacterial or viral infections
5. Pregnant or lactating patients
6. RhD antigen-negative patients
7. If the patient has clinically significant irregular antibodies, or has a history of clinically significant irregular antibodies in the past, and the availability of the relevant irregular antibody-negative blood products is limited (the presence or absence of clinical significance is determined by the " [Guidelines for red blood cell typing (erythrocyte testing), 4th edition (revised)]" [18]) Judge with reference to)
8. Patients for whom the principal investigator or co-investigator deems them inappropriate to participate in this study for any other reason

Table 1: Preoperative antiplatelet or anticoagulant withdrawal period

| Generic name | Withdrawal period |
| --- | --- |
| Antiplatelet agents | |
| Aspirin | 7 days |
| Clopidogrel | 5 days |
| Prasugrel | 7 days |
| Ticlopidine | 7 days |
| Ticagrerol | 3 days |
| Cilostazol | 3 days |
| Anticoagulants | |
| Warfarin | 3 days |
| Dabigatran | 3 days |
| Rivaroxaban | 3 days |
| Apixaban | 3 days |
| Edoxaban | 3 days |

＊For drugs with a discontinuation period of x days, fulfillment of the prescribed discontinuation period is defined as having elapsed more than x days (i.e., x × 24 hours) from the time of the last administration.

## **Rationale for Criteria**

### **Rationale for Inclusion Criteria**

1): Written consent is required as this is an interventional study

2–4) To identify patients suitable for inclusion in this trial based on the study's target population

### **Rationale for Exclusion Criteria**

1–3, 5) To ensure accurate evaluation of the study treatment
4, 6–8) To ensure participant safety

## **Selection and Exclusion Criteria for Registration**

Provisionally registered cases are registered after confirming that they meet the following inclusion criteria and do not conflict with the exclusion criteria.

## **Criterias of choice**

Patients who underwent allogeneic RBC transfusion in a heart-lung machine

## **Exclusion Criteria**

1. If the withdrawal period for antiplatelet or anticoagulants (see Table 1) was not complied with between the time of provisional registration and the date of surgery.
2. If the surgical procedure changes between the date of provisional registration and the date of surgery, or during surgery, and conflicts with the selection criteria for provisional registration, or meets any exclusion criteria for provisional registration.

## **Rationale for Criteria**

### **Rationale for Inclusion Criteria**

If this criterion is not met, surgery may proceed without transfusion, making the case outside the high-risk population targeted by this study. Bloodless surgery refers to a procedure in which anemia is corrected solely with salvaged RBC without the need for allogeneic RBC transfusion. This is only feasible when the blood loss is minimal.

### **Rationale for Exclusion Criteria**

Set to ensure valid evaluation of study treatment effects

# **Study Procedures**

## **Informed Consent**

### **Voluntary Participation**

This clinical study is conducted in accordance with the ethical principles of the Declaration of Helsinki and the Ethical Guidelines for Life Science and Medical Research Involving Human Subjects. Participation in this study is voluntary, and respect for the fundamental human rights of participants is maintained. Patients will receive appropriate medical care, regardless of whether they choose to participate in this study. The study will be fully explained to the participants using an explanatory document, and written informed consent will be obtained.

### **Procedure for Obtaining Informed Consent**

The principal investigator or sub-investigator explains the study using an explanatory document, provide sufficient time for consideration, and obtains written consent based on the participant’s free will.

When obtaining written consent, the participant must fully understand the content and sign and date the consent form. The investigator who provided the explanation also signs and dates this form.

### **Re-Consent**

If the study protocol is amended in a way that may affect the participant’s decision to continue the study, the revised explanatory document and consent form are approved by the ethics committee. Thereafter, the new content is explained to the participants, and re-consent is obtained in writing.

### **Withdrawal of Consent**

Participants may withdraw their consent at any time, without penalty. However, data already used in published research cannot be withdrawn. In cases where withdrawal is difficult owing to unforeseen circumstances, the matter is referred to the ethics committee for approval.

After withdrawal, no further data is collected, and any unused samples and data is not used in the future. The explanatory document includes information on the ability to withdraw, potential limitations, reasons, and contact information. When a participant expresses an intent to withdraw, it is recorded in the withdrawal form or medical record, and appropriate action is taken. If withdrawal is not possible, a clear explanation is provided to the participant to seek their understanding.

## **Provisional Registration**

The principal or sub-investigator obtains consent from the participant and verify that they meet the provisional registration inclusion criteria and do not meet any of the exclusion criteria. An identification number is then issued. If the patient does not meet the criteria, screening will end and regular medical care will be provided. The reason for ineligibility must be recorded in the screening logs.

Once the target sample size (142 final registrations) is reached, consent acquisition is temporarily suspended and resumed only if additional cases are required.

## **Final Registration**

Participants who meet the inclusion criteria and do not meet the exclusion criteria at the time of final registration are formally registered. Those deemed ineligible at this stage exit the study and receive standard medical care. In such cases, participants are informed of their ineligibility after surgery.

## **Randomization and Blinding**

After final registration, the principal or sub-investigator accesses the web-based system to input the required information for randomization. Participants are assigned in a 1:1 ratio to either the salvaged RBC group or the allogeneic RBC group using the stratified block randomization method.

Stratification factors include whether the surgery involves aortic arch replacement with antegrade cerebral perfusion and age (40–64 vs. 65 and older).

The study is single-blind: participants, outcome assessors, surgeons, surgical assistants, and physicians responsible for postoperative care remain blinded to the group assignments. Only the anesthesiologist involved in the procedure is unmasked. Blinding may be lifted by postoperative physicians in cases of uncontrollable bleeding that require knowledge of the assigned group..

## **Administration of the Study Treatment**

Consent and provisional registration are completed before the day of surgery. The final registration and randomization occurs during CPB. From the point of CPB weaning (protamine administration), the study product is administered based on the hemoglobin levels.

< Administration criteria for the study treatment>

If hemoglobin is less than 10.0 g/dL or is anticipated to drop below this level, transfusion is administered per group assignment.

### **Salvaged RBC Group (Investigational Product Group)**

From weaning off CPB until exiting the operating room, salvaged RBC is administered preferentially over allogeneic RBC to treat anemia. Residual blood in the CPB circuit (post-CPB blood) is collected immediately after the removal of the arterial and venous cannulas using the cell salvage device. If anemia is not corrected after administering all available salvaged RBC or if correction is not expected, allogeneic RBC is transfused.

If salvaged RBC become available during the transfusion of allogeneic RBC, the transfusion of allogeneic RBC is suspended and replaced with salvaged RBC. If salvaged RBC is unavailable when needed, allogeneic RBC is used.

After recovering the post-CPB blood, a 2 mL sample of the first returned salvaged RBC is collected and sent to an external laboratory to measure the heparin concentration.

After ICU admission, allogeneic RBC is used for the treatment of anemia.

### **Allogeneic RBC Group (Control Product Group)**

From CPB weaning until operating room exit, anemia is treated exclusively with allogeneic RBC without using salvaged RBC.

Residual blood in the CPB circuit (post-CPB blood) is still collected using the cell salvage device after removing the cannulas. However, the salvaged RBC is not transfused to the patient.

### **Criteria for Discontinuation of Study Treatment**

- In Salvaged RBC group, if purulent material or contamination from patient infection is observed in salvaged RBC, and its use may result in new infections
- In allogeneic RBC Group, if allogeneic RBC are insufficient and the surgeon or anesthesiologist determines that salvaged RBC is essential to avoid life-threatening anemia
- If unforeseen intraoperative complications require a change to a very high-risk surgical procedure
- If mechanical circulatory support becomes necessary during surgery
- If the principal investigator or sub-investigator deems continuation of study treatment to be difficult

## **Pre- and Concomintant Treatments**

### **Pre-Treatment Defined by the Study**

- Antithrombin III (Neuart®): If the ACT does not exceed 400 s after the administration of 300–500 U/kg of heparin before CPB initiation, administer 500–1500 U of antithrombin III.
- Prothrombin Complex Concentrate (Kcentra®): If PT-INR exceeds the upper limit of normal on the day of surgery despite appropriate warfarin withdrawal, administer 500–1000 U before surgery begins.

### **Concomintant Therapies**

- FFP: Begin after weaning from CPB. The ratio of FFP to total RBC transfusion volume (salvaged + allogeneic) should not exceed 1:1. If the fibrinogen level is ≥200 mg/dL and there is no bleeding tendency, FFP transfusion should not be administered.
- Cryoprecipitate: If fibrinogen is <100 mg/dL, administer the equivalent of 12 units of FFP. Consider 12 units of FFP if the fibrinogen level is 100–149 mg/dL.
- Tranexamic Acid: Administer a 1 g bolus at the start of surgery, followed by continuous infusion at 2 mg/kg/h until the end of surgery.
- Protamine: Administer 3 mg/kg at CPB weaning. If the clotting time ratio in a blood viscoelastic test (Quantra) is ≥1.2 or ACT exceeds H-ACT (ACT after heparin neutralization), additional doses are required.
- Voluven® (Colloid Solution): Limit the total intraoperative volume to 50 mL/kg.

### **Post-Treatment**

Within the first 12 hours after ICU admission, the transfusion protocols are as follows:

- RBC Transfusion: Administer allogeneic RBC if hemoglobin level is <10.0 g/dL or is expected to fall below this level. Leftover salvaged RBC should not be transfused. During re-thoracotomy within 12 hours, only allogeneic RBC is used for both groups. After 12 hours, there are no restrictions on the use of salvaged or allogeneic RBC during surgery.
- Platelet Transfusion: Administer if the platelet count is <100,000/μL and clinical bleeding is evident.
- FFP Transfusion: Administer if there is a bleeding tendency with APTT > 50 seconds, PT-INR > 1.3, or fibrinogen < 150 mg/dL. However, if the clotting time ratio is ≥ 1.2, protamine is administered, and the APTT value is reassessed thereafter. In cases of massive bleeding, transfusion is performed in the ICU with a target ratio of total FFP transfusion volume to total allogeneic RBC transfusion volume of approximately 1.
- Protamine: Administered if clotting time ratio ≥1.2 in the Quantra test or if ACT exceeds H-ACT.

## **Discontinuation Criteria and Procedures for Study Treatment**

### **Procedures for Discontinuation of Study Treatment**

If any discontinuation criteria are met, the principal or sub-investigator stops the administration of the investigational product. The date and reason for discontinuation are recorded in the medical charts. Data collection of the predefined observation items continues until the end of the study, even if the study treatment is discontinued. If discontinuation is due to an adverse event deemed likely to be caused by the study treatment, appropriate procedures are followed according to the adverse event reporting policy.

## **Discontinuation Criteria and Procedures for Study Participation**

### **Discontinuation Criteria**

If, for any of the following reasons, the subject is unable to complete the study or must discontinue the administration of the study formulation, the principal investigator (or co-investigator) terminates the subject's participation in the study.

1. The participant requests to withdraw from the study
2. Surgery is cancelled after consent has been obtained
3. For safety reasons, the principal investigator deems it necessary to discontinue participation
4. The study itself is terminated
5. Other reasons determined by the principal or sub-investigator

### **Procedures for Discontinuation of Participation**

If any discontinuation criteria are met, the principal investigator stops the study and record the discontinuation date and reason in the medical records. If the participant voluntarily withdraws from the study, no further data is collected from that point forward.

# **Observation and Examination Items and Schedule**

## **Visit 1. Consent Acquisition, Eligibility Confirmation, and Provisional Registration**

From 30 days before surgery to the day of surgery, the following data are collected from routine medical records to confirm eligibility and obtain consent.

| 1) Basic Subject Information | |
| --- | --- |
| Subject information | Date of obtaining consent, date of birth, age, gender |
| 2) Target Disease Information | |
| Disease information | Diagnosis, planned procedure, medical history, comorbidities |
| 3) Clinical and Laboratory Data**^*^** | |
| Physical findings | Height, weight, body surface area |
| Laboratory tests | Hemoglobin, platelet count, PT-INR, APTT, ABO and RhD blood type, presence of irregular antibodies |
| 4) Oral Medication Information | |
| Oral medecations | Use and withdrawal periods for steroids, immunosuppressants, antiplatelet and anticoagulant drugs |

- Laboratory results within 90 days prior to consent are used, with priority given to those closest to the consent date.

## **Visit 2. Final Registration and Randomization (Baseline)**

The following data are collected up to the time of CPB weaning on the day of surgery.

| 1) Clinical and Laboratory Data**^*2^** | |
| --- | --- |
| Physical findings | Height, weight, body surface area |
| Laboratory tests | White blood cell count, hemoglobin, platelet count, total protein, albumin, total bilirubin, direct bilirubin, AST, ALT, creatinine, LDH, CK, CK-MB, sodium, potassium, chloride, calcium, CRP, PT-INR, APTT, fibrinogen, D-dimer, FDP, ATIII activity |
| 2) Oral Medication Information | |
| Oral medications | Discontinuation period of antiplatelet agents and anticoagulants |
| 3) Surgical Information | |
| Treatment Information | Use of allogeneic RBC transfusion during CPB |

*2 Information are collected prior to the induction of general anesthesia. If results obtained during routine clinical care are available within 14 days before the day of surgery, those results are used.

## **Visit 3. During Study Treatment**

The following information is collected after weaning from CPB on the day of surgery (allowable range: +3 days). Data isobtained from the anesthesia records (ORSYS) and electronic medical records (MegaOak).

| 1) Study Treatment Information | |
| --- | --- |
| Treatment Information | - Volume of salvaged RBC and transfused allogeneic RBC (a) during from the start of general anesthesia to weaning from CPB, and (b) from weaning from CPB to leaving the operating room - Intraoperative blood loss - Heparin concentration in salvaged RBC (salvaged RBC group only)^*3^ |
| 2) Clinical and Laboratory Data |  |
| Vital signs | Blood pressure, heart rate, peripheral oxygen saturation (SpO₂), central venous pressure, pulmonary artery pressure, cardiac output (at the end of surgery) |
| Physical findings | Gross hematuria upon ICU admission |
| Blood gas analysis | Hemoglobin (at the following time points: after induction of general anesthesia, before initiation of CPB, during CPB, after CPB following protamine administration, and additionally as needed) |
| Laboratory tests | Hemoglobin, platelet count, PT-INR, APTT, fibrinogen, and blood viscoelastic testing (at the following time points: before and after weaning from CPB following protamine administration, at ICU admission, and additionally as needed)  Total protein, albumin, total bilirubin, direct bilirubin, AST ALT, creatinine, LDH, CK, CK-MB, sodium, potassium, chloride, calcium, and CRP (laboratory data collected at the time of ICU and subsequently as required). |
| 2) Concomitant Treatment Information | |
| Concomitant Therapy | - Volume of transfused FFP, platelet concentrates, and cryoprecipitate during (a) from the start of general anesthesia to weaning from CPB, and (b) from weaning from CPB to leaving the operating room - Total intraoperative doses of colloid solutions, tranexamic acid, heparin (excluding that used in the salvage system), and protamine |
| 3) Surgical Information | |
| Surgical Information | Definitive diagnosis, definitive surgical procedure, operative time, CPB time, aortic cross-clamp time, lowest temperature during CPB |
| 4) Adverse Events | |
| AEs | Date of onset, event name, known/unknown, seriousness, severity, causal relationship, treatment, outcome, date of outcome confirmation, other details |

*3 Measured for research purposes

## **Visit 4. End of Study**

Observations and examinations are performed 30 days after the initiation of study treatment (acceptable range: ±5 days). Data is collected from the critical care information system (AXIS) and electronic medical record system (MegaOak).

| 1) Study Treatment Information | |
| --- | --- |
| Treatment Information | - Postoperative chest tube blood loss within 12 hours after ICU admission - Total chest tube blood loss until removal - Transfusion volume within 24 hours after ICU admission (RBC, FFP, cryoprecipitate, platelet concentrates) - Duration of mechanical ventilation - Length of ICU stay - Postoperative hospital stay - Date of discharge |
| Discontinuation/interruption of treatment | Presence or absence; if applicable, record the date, reason, and subsequent management |
| 2) Clinical and Laboratory Data | |
| Laboratory tests  (obtained on the day after surgery) | White blood cell count, hemoglobin, platelet count, total protein, albumin, total bilirubin, direct bilirubin, AST, ALT, creatinine, LDH, CK, CK-MB, sodium, potassium, chloride, calcium, CRP, PT-INR, APTT, fibrinogen |
| 3) Adverse Event Reports | |
| AEs | Date of onset, event name, known or unknown, seriousness, severity, causal relationship, treatment, outcome, date of outcome confirmation, readmission status (presence/absence), date of readmission, date of discharge, and other relevant details |
| 4) Outcome Information | |
| Survival status | Outcome: alive, deceased, or lost to follow-up  Date of last confirmed survival or date of death; in case of death: cause of death If lost to follow-up: reason for discontinuation of follow-up |
| Stuidu withdrawal | Presence or absence; if applicable, record the date and reason for withdrawal |

## **When the study is stopped early (date of decision to discontinue+3)**

The following observations and tests are performed within three days of study discontinuation.

| 1) Study Treatment Information | |
| --- | --- |
| Discontinuation/interruption of treatment | Presence or absence; if applicable, record the date of discontinuation/interruption, reason, and subsequent management. |
| 2) Clinical and Laboratory Data ^*4^ | |
| Laboratory tests | White blood cell count, hemoglobin, platelet count, total protein, albumin, total bilirubin, direct bilirubin, AST, ALT, creatinine, LDH, CK, CK-MB, sodium, potassium, chloride, calcium, CRP, PT-INR, APTT, fibrinogen |
| 3) Adverse Event Information | |
| AEs | Date of onset, event name, known or unknown, seriousness, severity, causal relationship, treatment, outcome, date of outcome confirmation, readmission status (presence or absence), date of readmission, date of discharge, and other relevant details |
| 4) Outcome Information | |
| Survival status | Outcome: alive, deceased, or lost to follow-up  Date of last confirmed survival or date of death; in case of death: cause of death  If lost to follow-up: reason for loss to follow-up |
| Study withdrawal | If applicable, record the date of withdrawal and the reason |

*4 If test results from other visits or routine clinical care are available within one day prior to the date of study discontinuation, those results are used be used.**Observation and Inspection Schedule**

| Survey Period  Survey Items | Test Period | | | | Early discontinuation |
| --- | --- | --- | --- | --- | --- |
|  | Visit 1 | Visit 2 | Visit 3 | Visit 4 |  |
|  | **Consent, eligibility check, provisional registration** | **Final registration, allocation (prior to study treatment initiation)** | **During study treatment** | **End of Exam** |  |
| Stipulated days | -30 days~ | 0 days | 0 days | 30 days | ― |
| Tolerance | ― | ― | +3 days | ± 5 | +3 days |
| **Informmed consent** | ◎ |  |  |  |  |
| **Basic subject information** | ○ |  |  |  |  |
| **Target disease information** | ○ |  |  |  |  |
| **Study treatment information** |  |  | ○ | ○ | ○ |
| **Oral medication information** | ○ | ○ |  |  |  |
| **Physical findings** | ○^※1^ | ○^※2^ | ○ |  |  |
| **Vital signs** |  |  | ○ |  |  |
| **Laboratory tests (complete blood count, biochemistry, blood type-related, coagulation, viscoelastic testing)** | ○^※1^ | ○^※2^ | ○ | ○^※3^ | ○^※4^ |
| **Blood gas analysis** |  |  | ○ |  |  |
| **Heparin concentration in salvaged RBC**  **(Salvaged RBC group only)** |  |  | ● |  |  |
| **Concomitant treatment information** |  |  | ○ |  |  |
| **Surgery Information** |  | ○ | ○ |  |  |
| **Adverse events** |  |  |  |  |  |
| **Outcome information** |  |  |  | ○ | ○ |

　➡ : Assessed throughout the study period ◎: Mandatory items ○: Routine clinical examination item ●: Study-specific examination

*1 Results obtained from routine clinical care within 90 days before obtaining informed consent may be used for the study.

*2 Information must be collected before the induction of general anesthesia. If results from routine clinical care are available within 14 days before the day of surgery, they are used.

*3 Data obtained on the day after surgery is used.

*4 To be performed whenever possible. If results from other visits or routine clinical care are available within one day prior to the date of study discontinuation, they are used.

## **Definition of observation and inspection items**

### **Definition of the disease**

|  | definition |
| --- | --- |
| Myocardial infarction | A confirmed diagnosis of the above condition made by a cardiologist |
| sStroke | A confirmed diagnosis of the above condition made by a neurosurgeon or neurologist. |
| Heart failure | A history of hospitalization for the above condition, or classified as New York Heart Association Class II or higher, or with a left ventricular ejection fraction of less than 50% as determined by echocardiography. |
| Known coagulation and fibrinolytic disorders | Confirmed diagnoses of any of the following: idiopathic thrombocytopenic purpura, thrombotic thrombocytopenic purpura, aplastic anemia, antiphospholipid antibody syndrome, protein C deficiency, or other congenital or acquired coagulation or fibrinolytic disorders. |

# **Evaluation and Reporting of AEs**

## **Definition of AEs**

An AE refers to any untoward medical occurrence in a subject who has received the study treatment, regardless of whether a causal relationship with the study treatment can be established. A side effect is defined as an AE for which a causal relationship with the study product cannot be reasonably excluded.

## **Response to the Occurrence of AEs**

If an AE occurs, appropriate and optimal care must be provided promptly. For all AEs that occur during the study period, the following information must be promptly recorded in the medical record or related documents:
(1) name of the adverse event, (2) date of onset, (3) known or unknown event, (4) seriousness, (5) severity, (6) causal relationship with the study product, and (7) measures taken in response. Each AE must be assessed to determine whether it meets the criteria for an SAE. If judged to be an SAE, it is reported following the procedures described in Section 9.4.2.
In principle, all AEs judged to be causally related to the study product must be followed until resolution or stabilization, and the outcome and date of resolution must be documented.

## **Evaluation of AEs**

### **Definition of SAEs**

SAE are defined as any AE that meet one or more of the following criteria:

1. Results in death
2. Is life-threatening
3. Requires inpatient hospitalization or prolongation of existing hospitalization
4. Results in persistent or significant disability or incapacity
5. Is a medically important event that may jeopardize the subject
6. Other events that are equivalent in seriousness to those listed above
7. Congenital anomaly or birth defect in offspring

The following types of hospitalization are not considered SAEts under item 3, "hospitalization":

- Hospitalization solely for the purpose of conducting treatments or examinations that are scheduled prior to obtaining informed consent (e.g., scheduled surgeries or tests)
- Hospitalization for administrative or social reasons (e.g., receiving intravenous treatment, or cases where the subject's residence is far from the medical facility, making same-day treatment difficult)
- Hospitalization not intended for the treatment of an adverse event (e.g., hospitalization for examinations or health checkups)

### **Severity of AEs**

The severity of AEs in this study is classified as follows:

| Severity | definition |
| --- | --- |
| Mild | Symptoms or signs are recognized but do not interfere with daily activities, or are transient and recover without treatment, leaving no sequelae. |
| Moderate | Interferes with daily activities or requires medical intervention. |
| Severe | Significantly interferes with daily activities and requires surgical or other intensive intervention; may result in death or permanent dysfunction affecting daily life. |

### **Causal Relationship with the Study Product**

The principal investigator (or sub-investigator) must assess the causal relationship between the adverse event (AE) and the study product. The relationship should be classified as either **“Not ruled out”** or **“Ruled out.”** If the investigator cannot reasonably deny a causal relationship with the study product, the AE is assessed as **“Not ruled out.”**

## **Reporting of AEs**

### **Reporting Period for AEs**

AEs must be reported from the initiation of study product administration until 30 days thereafter or until the final study visit specified in the protocol, whichever is later. Any SAE for which a causal relationship with the study product cannot be ruled out must be reported **regardless of the reporting period**, as long as it occurs before the end of the study period.

### **Reporting Procedure for SAEs**

All personnel involved in the conduct of this study must follow the procedures below upon learning of an SAE in a study participant.

< Procedure >

1. The SAE is promptly reported to the principal investigator.
2. The principal investigator must report the SAE to the institutional ethics committee and study supervisor without delay.
3. The principal investigator also report the SAE to the head of the institution and take appropriate measures based on the protocol and institutional standard standard operating procedures. The relevant study personnel are informed of the events.
4. Upon receiving a response from the ethics committee, the principal investigator must report it to the institutional head and, if the committee provides specific recommendations, include the details of the corresponding actions in the report.
5. If the SAE is both unexpected and causally related to the study product, the principal investigator must report the incident and subsequent actions to the Ministry of Health, Labour, and Welfare of Japan using the designated format (Ethical Guidelines Form 3) and disclose the information on the institutional website.

## **Anticipated AEs**

The anticipated AEs associated with the administration of the study product are listed below.

### **Common AEs**

- Fever
- Hypothermia
- Hyperkalemia
- Dilutive coagulopathy
- Acidosis

### **Specific to Allogeneic RBC Transfusion**

- Hemolytic reactions
- Transfusion-associated dyspnea
- Allergic reactions
- Transfusion-related acute lung injury
- Transfusion-associated circulatory overload
- Post-transfusion purpura
- Graft-versus-host disease
- Transfusion-transmitted infections (viral, bacterial, protozoal, or other)
- Iron overload
- Hypocalcemia

### **Specific to Salvaged RBC Transfusion**

- Bacterial infections
- Hemolytic reactions

Other surgery-related complications not listed above may also occur and are recorded as adverse events.

# **Study Period**

Planned Study Period: From the date of ethical approval to March 31, 2030

Enrollment Period: From the date of ethical approval to September 30, 2026

Observation Period: From the date of ethical approval to November 4, 2026

# **Target Sample Size**

The target number of participants for final registration is 142 (71 in the salvaged RBC group and 71 in the allogeneic RBC group).

# **Statistics Considerations**

Statistical analysis is conducted in accordance with a separate document, the Statistical Analysis Plan.

# **Ethical Considerations**

## **Applicable Guidelines and Regulations**

This study is conducted in compliance with the ethical principles of the Declaration of Helsinki, Ethical Guidelines for Life Science and Medical Research Involving Human Subjects, and study protocol.

## **Procedures** **for Study Implementation**

Before initiating the study, the principal investigator must obtain approval from the institutional ethics committee and authorization from the head of the research institution.

## **Reporting to the Head of the Institution and Ethics Committee**

1) Reporting during the study

Researchers must report the following events to the principal investigator. In cases that fall under items 2 or 3, researchers must promptly report to the head of the research institution and, as needed, suspend, discontinue, or amend the study protocol (in accordance with Article 11, Sections 1, 2(2), and 2(3) of the Ethical Guidelines).

(1) Events or information that compromise or may compromise the ethical or scientific validity of the study

(2) Events or information that compromise or may compromise the integrity of the study implementation or the reliability of the study results

(3) Serious concerns related to human rights or the conduct of the study, such as the leakage of information related to the study

2) Periodic reporting

The principal investigator must submit an annual report to the ethics committee and the head of the institution regarding the progress of the study and the occurrence of adverse events.

3) Reporting at study completion (including premature termination)

The principal investigator must report the completion of the study to the ethics committee and the head of the institution in a timely manner.

## **Overall Assessment of Benefits and Burdens to Study Participants**

### **Benefits to participants and potential benefits of the study**

If this study demonstrates that the use of salvaged RBC during cardiovascular surgery does not increase postoperative bleeding, it may support the effective use of salvaged RBC that would otherwise be discarded and reduce the volume of allogeneic RBC transfusions. However, it is unlikely that the participants receives direct medical benefits during the study period.

### **Burden on study participants**

This study included blood sampling to measure heparin concentration in salvaged RBC for research purposes. However, only 2 mL of blood is collected from the processed salvaged RBC, which involves no risk of puncture and poses an extremely low risk of exacerbating anemia.

### **Anticipated risks to study participants**

There is a possibility of adverse events related to the study product, as described in Section 9.5. The risk of anemia due to blood sampling for research purposes is considered extremely low. Personal information is processed to prevent the immediate identification of individuals; thus, the risk of harm due to data leakage is considered to be low.

### **Measures to minimize risk**

Since personal information is processed, the risk of harm due to external data leakage is considered to be low.

### **Overall Evaluation**

The overall benefits of this study are considered to justify the potential risks and burdens of the participants.

### **Compensation for participation**

No compensation is provided to the participants for their involvement in this study.

## **Handling of inquiries from participants and their representatives**

The study information sheet includes the contact information (address, telephone number, etc.) of the study office as a point of contact for inquiries, and the office いis available to respond to questions or concerns from the participants and their representatives.

## **Preparation and Revision of the Study Information Sheet and Consent Form**

### **Preparation of documents and approval by the institution**

The principal investigator is responsible for preparing the study information sheets and consent forms. These documents must be submitted to the head of the institution and approved before the study initiation.

### **Required content of the study information sheet**

The study information sheet must include the following items, as specified in the Ethical Guidelines for Life Science and Medical Research Involving Human Subjects:

1. Title of the study and a statement that the head of the institution has approved its implementation
2. Names of cooperating institutions, names and affiliations of contributors providing existing specimens or data only, and names and affiliations of all principal investigators
3. Purpose and significance of the study
4. Methods and duration of the study, including the intended use of collected specimens and data
5. Reason for participant selection
6. Burden on participants, anticipated risks, and expected benefits
7. That participants may withdraw their consent at any time, and if there are limitations to such withdrawal, the reasons must be stated
8. That refusal or withdrawal of consent does not result in any disadvantageous treatment
9. Method of information disclosure about the study
10. That participants may request access to the study protocol and relevant information within the limits of protecting personal data and maintaining research originality, and how to make such requests
11. Handling of personal information, including details of anonymization or non-identifiable processing
12. Methods for storage and disposal of specimens and data
13. Funding sources and potential conflicts of interest of the institution or individual researchers
14. Handling of study results
15. How the study team responds to inquiries from participants or their representatives
16. Whether participants incur any financial burden or receive compensation
17. Information about alternative treatment options if the study involves procedures beyond standard care
18. Information about post-study care if the study involves procedures beyond standard care
19. Whether compensation is provided for study-related harm, and the details if applicable
20. If specimens or data may be used in unspecified future research or shared with other institutions, the means by which participants can confirm the content, the purpose, and the potential receiving institutions
21. If the study involves more than minimal invasion and includes interventions, a statement that study monitors, auditors, and the ethics committee may access relevant data and specimens for monitoring, auditing, and review purposes, provided participant confidentiality is protected

### **Revision of the study information sheet and consent form**

If, during the course of the study, the principal (or sub-) investigator obtains new information that may affect the participant’s consent, the study information sheet and consent form must be revised accordingly. Such new information may include newly recognized adverse events or the development of alternative treatments for the target disease. If the revisions are deemed to potentially affect the participant’s decision to continue, the revised documents must be submitted to the ethics committee for approval. After obtaining approval, participants must be re-informed, and new written consent must be obtained.

# **Protection of Personal Information**

To ensure the protection of the participants’ personal information, the following measures are observed in the conduct of this study:

1. When handling subject-related data, sufficient attention should be paid to confidentiality.
2. The personal information handled in this study is limited to what is necessary. The subject’s name is replaced with a study-specific identification number to prevent the immediate identification of individuals.
3. Anonymization is conducted promptly before starting the data analysis.
4. The correspondence table (linking subject names and ID numbers) is submitted to the Research Information Management Office prior to the start of data analysis, in accordance with the institutional policy on personal information management and data protection. Until then, it is stored in a locked drawer of a personal desk in the department under the responsibility of the principal investigator.

# **Data Management**

## **Preparation of CRF**

The principal (or sub-) investigator or study collaborators is enter the observation and assessment data into the CRF. The contents of the CRFs must be consistent with source documents. Data is entered and managed using Microsoft Excel.

## **Amendments or Corrections to the CRFs**

If amendments or corrections to the CRFs are necessary, the responsible personnel do so in accordance with the CRF amendment/correction procedures.

## **Attribution of collected data**

All information obtained in this study belongs to the National Cerebral and Cardiovascular Center and may not be accessed without the principal investigator’s permission.

# **Storage of Samples and Data**

## **Method of Storage**

The following procedures are followed for the storage and management of the samples and data:

1. Electronic study data is stored under the responsibility of the principal investigator in accordance with the information security policy of the National Cerebral and Cardiovascular Center, Japan. Specifically, PCs and storage devices in the department are protected as follows.
   1. Installation and regular updating of antivirus software
   2. Access control using IDs and passwords, allowing access only to authorized research personnel
2. Blood samples for testing are stored in a locked freezer in the equipment room in front of Operating Room 10, under the responsibility of the principal investigator, until they are sent to the commissioned laboratory for analysis.

## **Transfer of Samples/Data to External Organizations**

Transfer of samples/data: Not applicable

## **Retention Period of samples and information**

According to the internal regulations of the National Cerebral and Cardiovascular Center regarding research misconduct, data is retained for 10 years following publication. Residual samples are not stored, as none remain. After the retention period, paper records are shredded or dissolved, electronic data is be deleted, and storage media is physically destroyed to ensure complete anonymization.

## **Future Use of Data**

The samples and data collected in this study is used for the purposes of this study. If future use or sharing for other research arises, it is conducted only after the development of a new protocol and approval by the ethics committee and institution.

# **Quality Control & Quality Assurance**

## **Compliance, Deviations, and Amendments**

The principal (or sub-) investigator and study collaborators must comply with the study protocol in conducting this research. No deviations or changes to the protocol are made without prior approval from the ethics committee. All deviations are documented. However, deviations or changes may be permitted if they are deemed medically necessary.

## **Protocol Amendments**

If new information necessitating a protocol amendment arises, the principal investigator revises the protocol and prepare a record of the revisions and their rationale. Ethics committee approval and institutional permission must be obtained before the implementation. If patient enrollment needs to be suspended during the revision process, the principal investigator must notify the study collaborators accordingly.

## **Monitoring**

Monitoring is conducted by a monitor designated by the principal investigator to assess the study’s framework and implementation. The details are described in a separate monitoring procedure.

## **Audit**

No audit is conducted, as the study is funded solely by the internal research and development funds of the National Cerebral and Cardiovascular Center.

# **Termination or Discontinuation of the Study**

## **Study Termination**

When all analyses and manuscript submissions are completed, the principal investigator notifies the study collaborators of the study’s completion and performs all related closing procedures, including reporting to the head of the institution.

## **Discontinuation or Suspension of the Study**

### **Criteria for Discontinuation or Suspension**

The principal investigator may discontinue or suspend the study if any of the following criteria are met. Notification of discontinuation must be submitted to the ethics committee within 10 days of the decision.

1. Occurrence of serious adverse events or other safety concerns making continuation of the study difficult
2. New information from publications or academic meetings indicates that the study is no longer feasible or meaningful
3. Serious noncompliance with ethical guidelines or related regulations makes continuation difficult
4. Significant delays in enrollment prevent successful completion of the study
5. Any other reason the principal investigator deems discontinuation necessary

### **Procedures for Discontinuation or Suspension**

If the principal investigator determines that the study should be discontinued or suspended based on the above criteria, the following procedures are followed.

1. The principal investigator promptly informs all personnel involved in the decision and the reasons for discontinuation or suspension.
2. The principal investigator promptly notifies affected participants and take appropriate action. In addition, the principal investigator reports the decision to the head of the institution and related departments and follow the prescribed institutional procedures.

# **Funding and Conflict of Interest**

## **Funding**

This study is self-funded by the National Cerebral and Cardiovascular Center. The funding source is not be involved in the design, management, analysis, or interpretation of this study.

## **Conflict of Interest**

This study is funded by the 2023 fiscal year budget for cardiovascular disease research and development. No commercial entity is involved, and there is no conflict of interest to disclose.
Any potential conflict of interest must be reported to the Conflict of Interest Committee for Medical Research and approved by it. This study ensures that no disadvantage arises for the participants as a result of any conflict of interest.

# **Compensation for Study-related Injury**

If a study-related injury occurs, the institution provides appropriate and optimal care to the participant. As this study involves the use of blood products that are already in routine clinical use, any adverse reactions are treated as part of standard care, and no additional compensation (e.g., reimbursement of out-of-pocket expenses, condolence payments, or disability compensation) is provided. In applicable cases, compensation may be sought through the pharmaceutical adverse reaction relief system.

No clinical trial insurance is purchased for this study.

# **Post-study Medical Care for Participants**

After study completion, the participants continue to receive treatment under standard insurance-based care.

# **Handling of Study Results and Health Information**

Participants are informed that this study is not expected to yield new, individual health-related results.

# **Publication and Ownership of Study Results**

## **Study Registration**

Before the enrollment of the first participant begins, the study overview is registered in the Japan Registry of Clinical Trials, and the progress and results are updated accordingly.

## **Publication of Study Results**

After the study is completed, the results are published in academic journals or presented at conferences by the designated authors. Prior to publication, the authors consult with the principal investigator and other stakeholders and obtain approval. The approval number for the study will be included in all publications.

## **Ownership of Study Data and Results**

Intellectual property arising from this study, in principle, belongs to the institution, in accordance with internal regulations on employee inventions. The ownership does not extend to the participants or their families.

# **Study Implementation Structure**

See the attached document: Study Implementation Structure.

# **Oversight of Contracted Organizationss**

Outsourcing: Yes

1. Contract organization: SRL
2. Provided item: Measurement of heparin concentration
3. Details of contracted service: Measurement of heparin concentration in salvaged RBC
4. Oversight method: Compliance with the contract is checked as required. Any identified issues are addressed accordingly.

# **References**

1. Tanaka J, Shikano C, Akita T, Sugiyama A, Kurisu A. [Future projections of blood donation demand and supply] Health and Labour Sciences Research Grant (Regulatory Science for Pharmaceuticals and Medical Devices Policy Research Project), FY2020 Research Report. 2020. [in Japanese]

2. Matoba M, Akashi Y, Kosaka S, Sakata Y, Takeishi Y, Tsutsui H, et al. [Report of the survey on clinical practice in cardiovascular disease (2020 implementation and publication)] 2020. Available from: <https://www.j-circ.or.jp/jittai_chosa/media/jittai_chosa2019web_ver2_revise20241015.pdf>. [in Japanese]

3. Japan Society of Transfusion Medicine and Cell Therapy SoCSoTP. [Survey on the actual use of blood products, FY2019 report] 2019. Available from: <https://yuketsu.jstmct.or.jp/wp-content/uploads/2020/09/d888ba7e81de8e35f4fc1d1158f9a050.pdf>. [in Japanese]

4. Zacharias A, Habib RH. Factors predisposing to median sternotomy complications. Deep vs superficial infection. Chest. 1996;110(5):1173–8. doi: 10.1378/chest.110.5.1173. PubMed PMID: 8915216.

5. Leal-Noval SR, Marquez-Vácaro JA, García-Curiel A, Camacho-Laraña P, Rincón-Ferrari MD, Ordoñez-Fernández A, et al. Nosocomial pneumonia in patients undergoing heart surgery. Critical care medicine. 2000;28(4):935–40. doi: 10.1097/00003246-200004000-00004. PubMed PMID: 10809262.

6. Ranucci M, Pavesi M, Mazza E, Bertucci C, Frigiola A, Menicanti L, et al. Risk factors for renal dysfunction after coronary surgery: the role of cardiopulmonary bypass technique. Perfusion. 1994;9(5):319–26. doi: 10.1177/026765919400900503. PubMed PMID: 7833539.

7. Engoren MC, Habib RH, Zacharias A, Schwann TA, Riordan CJ, Durham SJ. Effect of blood transfusion on long-term survival after cardiac operation. The Annals of thoracic surgery. 2002;74(4):1180–6. doi: 10.1016/s0003-4975(02)03766-9. PubMed PMID: 12400765.

8. Dalrymple-Hay MJ, Pack L, Deakin CD, Shephard S, Ohri SK, Haw MP, et al. Autotransfusion of washed shed mediastinal fluid decreases the requirement for autologous blood transfusion following cardiac surgery: a prospective randomized trial. Eur J Cardiothorac Surg. 1999;15(6):830–4. doi: 10.1016/s1010-7940(99)00112-8. PubMed PMID: 10431866.

9. Wong JC, Torella F, Haynes SL, Dalrymple K, Mortimer AJ, McCollum CN. Autologous versus allogeneic transfusion in aortic surgery: a multicenter randomized clinical trial. Ann Surg. 2002;235(1):145–51. doi: 10.1097/00000658-200201000-00019. PubMed PMID: 11753054; PubMed Central PMCID: PMCPMC1422408.

10. Niranjan G, Asimakopoulos G, Karagounis A, Cockerill G, Thompson M, Chandrasekaran V. Effects of cell saver autologous blood transfusion on blood loss and homologous blood transfusion requirements in patients undergoing cardiac surgery on- versus off-cardiopulmonary bypass: a randomised trial. Eur J Cardiothorac Surg. 2006;30(2):271–7. Epub 20060707. doi: 10.1016/j.ejcts.2006.04.042. PubMed PMID: 16829083.

11. Shen S, Zhang J, Wang W, Zheng J, Xie Y. Impact of intra-operative cell salvage on blood coagulation in high-bleeding-risk patients undergoing cardiac surgery with cardiopulmonary bypass: a prospective randomized and controlled trial. Journal of Translational Medicine. 2016;14(1). doi: 10.1186/s12967-016-0986-6.

12. Lloyd TD, Geneen LJ, Bernhardt K, McClune W, Fernquest SJ, Brown T, et al. Cell salvage for minimising perioperative allogeneic blood transfusion in adults undergoing elective surgery. Cochrane Database Syst Rev. 2023;9(9):Cd001888. Epub 20230908. doi: 10.1002/14651858.CD001888.pub5. PubMed PMID: 37681564; PubMed Central PMCID: PMCPMC10486190.

13. Tomoko A, Koji K, Ikuko O, Kinari T, Seiko S, Saeko O, et al. INTRAOPERATIVE AUTOLOGOUS BLOOD COLLECTION AND AUTOTRANSFUSION FOR THE REDUCTION OF ALLOGENIC BLOOD TRANSFUSION CARDIOVASCULAR SURGERY. Japanese Journal of Transfusion and Cell Therapy. 2017;63(5):674–82.

14. Pagano D, Milojevic M, Meesters MI, Benedetto U, Bolliger D, von Heymann C, et al. 2017 EACTS/EACTA Guidelines on patient blood management for adult cardiac surgery. Eur J Cardiothorac Surg. 2018;53(1):79–111. doi: 10.1093/ejcts/ezx325. PubMed PMID: 29029100.

15. Tibi P, McClure RS, Huang J, Baker RA, Fitzgerald D, Mazer CD, et al. STS/SCA/AmSECT/SABM Update to the Clinical Practice Guidelines on Patient Blood Management. The Annals of thoracic surgery. 2021;112(3):981–1004. Epub 20210630. doi: 10.1016/j.athoracsur.2021.03.033. PubMed PMID: 34217505.

16. Horan TC, Andrus M, Dudeck MA. CDC/NHSN surveillance definition of health care-associated infection and criteria for specific types of infections in the acute care setting. Am J Infect Control. 2008;36(5):309–32. doi: 10.1016/j.ajic.2008.03.002. PubMed PMID: 18538699.

17. Khwaja A. KDIGO clinical practice guidelines for acute kidney injury. Nephron Clin Pract. 2012;120(4):c179–84. Epub 20120807. doi: 10.1159/000339789. PubMed PMID: 22890468.

18. Okuda M, Ikemoto J, Ishimaru K, Uchikawa M, Kajiwara M, Kitazawa J, et al. [Guidelines for red blood cell typing (erythrocyte testing), 4th edition (revised)]. Journal of the Japan Society of Transfusion and Cell Therapy. 2022;68:539–56. [in Japanese]
