# Supplementary material for: Intraoperative cell-salvaged versus allogeneic red blood cell transfusions in high-bleeding-risk cardiovascular surgery: Protocol for a single-center, randomized, parallel-group, noninferiority trial
Source: PLoS One. 2025 Oct 24;20(10):e0334397. doi: 10.1371/journal.pone.0334397 (PMC12551814; doi:10.1371/journal.pone.0334397)
Supplement: S3 File — (DOCX) [file pone.0334397.s003.docx]

心臓大血管手術における回収式自己血輸血と同種赤血球輸血の術後出血量に与える影響の比較：ランダム化比較非劣性試験

**S**tudy on the **E**ffect of intraoperative cell-salvaged versus allogeneic transfusion on postoperative blood **L**oss and coagulation **F**unction (SELF試験)

研究責任者：月永　晶人　麻酔科

第1.1版　2024年7月11日

第1.2版　2024年9月13日

第1.3版　2024年12月4日

第1.4版　2025年8月12日

第1.5版　2025年10月1日

| **倫理審査を受けた試験実施計画書の遵守**  本試験は、ヘルシンキ宣言に基づく倫理原則及び「人を対象とする生命科学・医学系研究に関する倫理指針」に従い、被験者の基本的人権を尊重し、倫理審査委員会の審査及び研究機関の長の許可を受けた試験実施計画書を遵守して実施される。 |
| --- |

# **0. 試験概要**

| 試験課題名 | | 心臓大血管手術における回収式自己血輸血と同種赤血球輸血の術後出血量に与える影響の比較：ランダム化比較非劣性試験 |
| --- | --- | --- |
| 研究責任者 | | 月永　晶人 |
| 試験の目的 | | 出血リスクの高い人工心肺を用いた心臓大血管手術の、人工心肺離脱後の貧血に対して回収式自己血を輸血することは、回収式自己血を使用せずに同種赤血球のみを輸血する輸血療法と比較して、ICU入室後出血量が多くならないという仮説を、単盲検ランダム化比較非劣性試験で評価することを目的とする。 |
| 試験デザイン | | 単盲検、ランダム化、並行群間、非劣性検証試験 |
| 対象患者 | | 人工心肺を使用する心臓大血管手術を受ける患者 |
| 適格基準 | 選択基準 | ＜仮登録時＞   1. 本人から同意の得られた患者 2. 同意取得時の年齢が40歳以上の患者 3. 人工心肺を使用し、胸骨正中切開アプローチによる心臓大血管手術を待機的に受ける患者 4. 以下のいずれかに該当する患者（出血リスクの高い症例に該当する）    1. 過去に胸骨正中切開アプローチによる心臓大血管手術の既往がある再開胸手術    2. 大動脈基部手術（ロス手術、弁輪拡大を伴う大動脈弁置換術、大動脈基部置換術）、上行大動脈置換術、弓部大動脈置換術のいずれかを行う患者    3. 三尖弁輪形成術を除く2弁以上の弁膜症手術を行う患者    4. 冠動脈バイパス手術に三尖弁輪形成術を除く弁膜症手術を合わせて行う患者   ＜本登録時＞  人工心肺中に同種血輸血を実施した患者 |
|  | 除外基準 | ＜仮登録時＞   1. 下行大動脈置換術、胸腹部大動脈置換術、心臓移植術、補助人工心臓移植術、肺動脈弁置換術のいずれかを受ける患者 2. 出血リスクが極めて高いと考えられる患者 3. 術前の抗血小板薬または抗凝固薬の休薬期間を遵守していない患者 4. 活動性の細菌あるいはウイルス感染症を合併している患者 5. 妊娠中または授乳中の患者 6. RhD抗原陰性患者 7. 不規則抗体陽性患者 8. その他の理由により、研究責任者または分担者が本試験への参加を不適当と判断した患者   ＜本登録時＞   1. 仮登録後から手術日までの間に、抗血小板薬または抗凝固薬の休薬期間（表1参照）が遵守されていなかった場合 2. 仮登録後から手術日までの間に、あるいは手術中に手術術式が変更し、仮登録の選択基準から抵触するか、仮登録のいずれかの除外基準を満たした場合 |
| 試験製剤 | 回収式自己血群（被験製剤） | 人工心肺離脱後の貧血に対して、回収式自己血を同種赤血球製剤に優先して使用する。回収式自己血を使いきってもなお貧血が是正されていない場合、あるいは是正されていないことが想定される場合は同種赤血球製剤を輸血する。 |
|  | 同種赤血球製剤群（対照製剤） | 人工心肺離脱後の貧血に対して、回収式自己血を使用せず、同種赤血球製剤のみ輸血する。 |
| 試験の実施手順の概要 | | 手術前に同意取得・仮登録し、手術中に本登録を行う。本登録後、被験者は回収式自己血群あるいは同種赤血球輸血群のいずれかに1:1の割合で無作為に割り付けられる。割り付けは層別ブロック法を用いる。本試験は、被験者、評価者、執刀医と手術助手、術後管理に関わる医師に対して、試験期間を通じて盲検性が保持されるよう実施する。試験製剤は血液検査でヘモグロビン10.0g/dL未満の貧血を確認した、あるいは想定される際に投与する。 |
| 試験製剤の投与期間 | | 人工心肺離脱時から手術室退室時まで |
| 評価項目 | 主要  評価項目 | ICU入室後12時間のドレーン排液総量 |
|  | 副次  評価項目 | 1. 全身麻酔開始時からICU入室後12時間までの同種赤血球輸血量 2. ICU入室後48時間までの再開胸止血術あるいは再開胸血腫除去術の発生割合 3. ICU入室後12時間までのドレーン排液総量が1000mLを超える割合 |
|  | 安全性評価項目 | 1. 術後感染症の発生割合 2. ICU入室時の肉眼的血尿の有無の割合 3. 手術後7日以内の急性腎障害の発症割合 |
| 予定登録症例数 | | 142例（回収式自己血使用群 71例　同種血使用群 71例） |
| 症例数の設定根拠 | | 本研究の適格基準を満たす患者の予備データで、同種赤血球製剤のみを用いた59例のICU入室後12時間のドレーン排液量の平均値674mL、標準偏差400mLであった。α=0.025、β=0.2、非劣勢マージンを既報 を参考にして200ｍLとして、両群で128名（1群64例）が算出された。脱落率10%とし両群で142名（1群71例）を設定した。 |
| 予定実施期間 | | 試験予定期間：研究許可日～2030年3月31日  症例登録期間：研究許可日～2026年9月30日  症例観察期間：研究許可日～2026年11月4日 |
| 試験事務局 | | 国立循環器病研究センター　輸血管理部  住所：〒564-85655 大阪府吹田市岸部新町6-1  TEL：06-6170-1069 内線40563　FAX：06-6170-1953  e-mail：onishi.yuko@ncvc.go.jp |

出血リスクの高い人工心肺を用いた心臓大血管手術を待機的に受ける患者

同意取得

適格性の確認・仮登録

人工心肺中の同種赤血球輸血

実施せず

実施

終了

本登録・ランダム化割付

観察期間：30日間

主要評価項目としてICU入室後12時間のドレーン排液総量を測定する。副次評価項目のために試験治療後30日間患者を追跡する

　同種赤血球製剤群

同種赤血球輸血のみを行う

投与期間：人工心肺離脱時から手術室退室まで

回収式自己血群

回収式自己血を同種赤血球製剤に優先して投与

投与期間：人工心肺離脱時から手術室退室まで

略語一覧

| ACT | Activated clotting time | 活性化凝固時間 |
| --- | --- | --- |
| ALT | Alanine aminotransferase | アラニンアミノ基転移酵素 |
| APTT | Activated partial thromboplastin time | 活性化部分トロンボプラスチン時間 |
| AST | Aspartate aminotransferase | アスパラギン酸アミノ基転移酵素 |
| ATIII | Anti thrombin III | アンチトロンビンIII |
| BNP | Brain natriuretic peptide | 脳性ナトリウム利尿ペプチド |
| CDC | Centers for disease control and prevention | アメリカ疾病予防管理センター |
| CK | Creatine kinase | クレアチンキナーゼ |
| CRP | C-reactive protein | C反応性蛋白 |
| FAS | Full analysis set | 最大の解析対象集団 |
| FBS | Fast blood sugar | 空腹時血糖 |
| FDP | Fibrin/fibrinogen degradation products | フィブリン・フィブリノゲン分解産物 |
| ICU | Intensive care unit | 集中治療室 |
| ITT | Intent-to-treat population | 治療意図の原理による解析対象集団 |
| LDH | Lactate dehydrogenase | 乳酸脱水素酵素 |
| PPS | Per protocol set | 試験実施計画書に適合した解析対象集団 |
| PT-INR | Prothrombin time-international normalized ratio | プロトロンビン時間国際標準化比 |
| SSI | Surgical site infection | 手術部位感染 |

目次

[**0. 試験概要** i](#_Toc210223404)

[**1.** **背景と論理的根拠** 1](#_Toc210223405)

[**1.1.** **国内外における対象疾患の現状** 1](#_Toc210223406)

[**1.2.** **これまでに実施されてきた標準治療の経緯及び内容** 1](#_Toc210223407)

[**1.3.** **現在の標準治療の内容及び治療成績** 1](#_Toc210223408)

[**1.4.** **当該臨床試験の必要性につながる、現在の標準治療の課題、不明点など** 1](#_Toc210223409)

[**1.5.** **試験実施の妥当性** 2](#_Toc210223410)

[**2.** **目的** 2](#_Toc210223411)

[**3.** **試験デザイン** 2](#_Toc210223412)

[**4.** **評価項目** 2](#_Toc210223413)

[**4.1.** **主要評価項目** 2](#_Toc210223414)

[**4.2.** **副次評価項目** 3](#_Toc210223415)

[**4.3.** **安全性評価項目** 3](#_Toc210223416)

[**4.4.** **探索的評価項目** 3](#_Toc210223417)

[**5.** **試験製剤情報** 4](#_Toc210223418)

[**5.1.** **被験製剤** 4](#_Toc210223419)

[**5.1.1.** **名称・含量等** 4](#_Toc210223420)

[**5.1.2.** **包装・表示等** 4](#_Toc210223421)

[**5.1.3.** **品質管理** 4](#_Toc210223422)

[**5.1.4.** **回収から返血までの流れ** 4](#_Toc210223423)

[**5.1.5.** **Cell Saver Eliteの設定** 4](#_Toc210223424)

[**5.2.** **対照製剤** 5](#_Toc210223425)

[**5.2.1.** **名称・含量等** 5](#_Toc210223426)

[**5.2.2.** **包装・表示等** 5](#_Toc210223427)

[**5.2.3.** **品質管理** 5](#_Toc210223428)

[**6.** **対象** 5](#_Toc210223429)

[**6.1.** **仮登録の選択・除外基準** 5](#_Toc210223430)

[**6.1.1.** **選択基準** 5](#_Toc210223431)

[**6.1.2.** **除外基準** 5](#_Toc210223432)

[**6.1.3.** **設定根拠** 6](#_Toc210223433)

[**6.1.3.1.** **選択基準の設定根拠** 6](#_Toc210223434)

[**6.1.3.2.** **除外基準の設定根拠** 6](#_Toc210223435)

[**6.2.** **本登録の選択・除外基準** 6](#_Toc210223436)

[**6.2.1.** **選択基準** 7](#_Toc210223437)

[**6.2.2.** **除外基準** 7](#_Toc210223438)

[**6.2.3.** **設定根拠** 7](#_Toc210223439)

[**6.2.3.1.** **選択基準の設定根拠** 7](#_Toc210223440)

[**6.2.3.2.** **除外基準の設定根拠** 7](#_Toc210223441)

[**7.** **試験の実施手順** 7](#_Toc210223442)

[**7.1.** **インフォームド・コンセント** 7](#_Toc210223443)

[**7.1.1.** **試験参加の任意性** 7](#_Toc210223444)

[**7.1.2.** **インフォームド・コンセントを受ける手続き** 7](#_Toc210223445)

[**7.1.3.** **再同意** 7](#_Toc210223446)

[**7.1.4.** **同意の撤回** 8](#_Toc210223447)

[**7.2.** **被験者の仮登録** 8](#_Toc210223448)

[**7.3.** **被験者の本登録** 8](#_Toc210223449)

[**7.4.** **割り付けと盲検化** 8](#_Toc210223450)

[**7.5.** **試験製剤の投与** 9](#_Toc210223451)

[**7.5.1.** **回収式自己血群（被験製剤群）** 9](#_Toc210223452)

[**7.5.2.** **同種赤血球製剤群（対照製剤群）** 9](#_Toc210223453)

[**7.5.3.** **試験治療の中止基準** 9](#_Toc210223454)

[**7.6.** **前治療および併用療法** 10](#_Toc210223455)

[**7.6.1.** **研究として定める前治療** 10](#_Toc210223456)

[**7.6.2.** **併用療法** 10](#_Toc210223457)

[**7.6.3.** **後治療** 10](#_Toc210223458)

[**7.7.** **試験治療の中止基準およびその手順** 11](#_Toc210223459)

[**7.7.1.** **試験製剤投与の中止手順** 11](#_Toc210223460)

[**7.8.** **被験者の試験参加の中止基準およびその手順** 11](#_Toc210223461)

[**7.8.1.** **被験者の試験参加の中止基準** 11](#_Toc210223462)

[**7.8.2.** **被験者の試験参加の中止手順** 11](#_Toc210223463)

[**8.** **観察・検査項目とスケジュール** 11](#_Toc210223464)

[**8.1.** **Visit 1. 同意取得・適格性確認・仮登録** 11](#_Toc210223465)

[**8.2.** **Visit 2. 本登録・割り付け（ベースライン）** 12](#_Toc210223466)

[**8.3.** **Visit 3. 試験治療中** 12](#_Toc210223467)

[**8.4.** **Visit 4. 試験終了時** 13](#_Toc210223468)

[**8.5.** **試験早期中止時　（中止決定日＋3）** 14](#_Toc210223469)

[**8.6.** **観察・検査項目の定義** 15](#_Toc210223470)

[**8.6.1.** **疾患の定義** 15](#_Toc210223471)

[**9.** **有害事象の評価・報告** 16](#_Toc210223472)

[**9.1.** **有害事象の定義** 16](#_Toc210223473)

[**9.2.** **有害事象発現時の対応** 16](#_Toc210223474)

[**9.3.** **有害事象の評価** 16](#_Toc210223475)

[**9.3.1.** **重篤な有害事象の定義** 16](#_Toc210223476)

[**9.3.2.** **有害事象の重症度** 17](#_Toc210223477)

[**9.3.3.** **試験製剤との因果関係** 17](#_Toc210223478)

[**9.4.** **有害事象の報告** 17](#_Toc210223479)

[**9.4.1.** **有害事象の報告期間** 17](#_Toc210223480)

[**9.4.2.** **重篤な有害事象の報告手順** 17](#_Toc210223481)

[**9.5.** **予測される有害事象等** 18](#_Toc210223482)

[**9.5.1.** **共通** 18](#_Toc210223483)

[**9.5.2.** **同種赤血球製剤に特異的なもの** 18](#_Toc210223484)

[**9.5.3.** **回収式自己血輸血に特異的なもの** 18](#_Toc210223485)

[**10.** **試験期間** 18](#_Toc210223486)

[**11.** **目標症例数** 18](#_Toc210223487)

[**12.** **統計的事項** 18](#_Toc210223488)

[**13.** **倫理的事項** 19](#_Toc210223489)

[**13.1.** **遵守すべき諸規則** 19](#_Toc210223490)

[**13.2.** **試験実施の手続き** 19](#_Toc210223491)

[**13.3.** **研究機関の長及び倫理審査委員会への報告** 19](#_Toc210223492)

[**13.4.** **試験参加に伴う被験者の利益と不利益の総合的評価** 19](#_Toc210223493)

[**13.4.1.** **試験参加に伴う被験者の利益および試験がもたらす利益** 19](#_Toc210223494)

[**13.4.2.** **試験参加に伴う被験者の負担** 19](#_Toc210223495)

[**13.4.3.** **試験参加に伴う被験者への予測されるリスク** 19](#_Toc210223496)

[**13.4.4.** **被験者のリスクを最小化するための対策** 20](#_Toc210223497)

[**13.4.5.** **総合評価** 20](#_Toc210223498)

[**13.4.6.** **試験参加に伴う被験者への謝礼等** 20](#_Toc210223499)

[**13.5.** **被験者及びその関係者からの相談等への対応** 20](#_Toc210223500)

[**13.6.** **説明文書・同意文書の作成と改訂** 20](#_Toc210223501)

[**13.6.1.** **説明文書の作成および研究機関の長の許可** 20](#_Toc210223502)

[**13.6.2.** **説明文書への記載事項** 20](#_Toc210223503)

[**13.6.3.** **説明文書・同意文書の改訂** 21](#_Toc210223504)

[**14.** **個人情報の保護** 21](#_Toc210223505)

[**15.** **データの管理** 22](#_Toc210223506)

[**15.1.** **症例報告書の作成** 22](#_Toc210223507)

[**15.2.** **症例報告書の変更・修正** 22](#_Toc210223508)

[**15.3.** **収集したデータの帰属** 22](#_Toc210223509)

[**16.** **試料・情報の保存** 22](#_Toc210223510)

[**16.1.** **試料・情報の保管方法** 22](#_Toc210223511)

[**16.2.** **外部の機関との試料・情報の授受** 22](#_Toc210223512)

[**16.3.** **試料・情報の保存期間** 23](#_Toc210223513)

[**16.4.** **将来の研究への情報の利用** 23](#_Toc210223514)

[**17.** **品質管理と品質保証** 23](#_Toc210223515)

[**17.1.** **試験実施計画書の遵守・逸脱・変更** 23](#_Toc210223516)

[**17.2.** **試験実施計画書の改訂** 23](#_Toc210223517)

[**17.3.** **モニタリング** 23](#_Toc210223518)

[**17.4.** **監査** 23](#_Toc210223519)

[**18.** **試験全体の終了もしくは中止** 23](#_Toc210223520)

[**18.1.** **試験全体の終了** 23](#_Toc210223521)

[**18.2.** **試験全体の中止・中断** 24](#_Toc210223522)

[**18.2.1.** **試験全体の中止・中断基準** 24](#_Toc210223523)

[**18.2.2.** **試験全体の中止・中断手順** 24](#_Toc210223524)

[**19.** **試験の資金源及び利益相反** 24](#_Toc210223525)

[**19.1.** **試験の資金源** 24](#_Toc210223526)

[**19.2.** **利益相反** 24](#_Toc210223527)

[**20.** **健康被害の補償** 25](#_Toc210223528)

[**21.** **試験実施後における被験者への医療の提供に関する対応** 25](#_Toc210223529)

[**22.** **試験により得られた情報等の取り扱い** 25](#_Toc210223530)

[**23.** **試験結果の公表と成果の帰属** 25](#_Toc210223531)

[**23.1.** **試験の登録** 25](#_Toc210223532)

[**23.2.** **試験成果の公表** 25](#_Toc210223533)

[**23.3.** **試験成果及びデータの帰属** 25](#_Toc210223534)

[**24.** **試験の実施体制** 25](#_Toc210223535)

[**25.** **業務委託先の監督方法** 25](#_Toc210223536)

[**26.** **参考文献** 26](#_Toc210223537)

# **背景と論理的根拠**

## **国内外における対象疾患の現状**

少子高齢化が進む日本では、献血者数の減少および高齢者人口の増加に伴う血液製剤需要の増加が見込まれており、2025年には33-65万人分の献血が不足すると予想されている[1]。したがって、血液製剤の使用量を削減することは重要な課題である。日本では、心臓血管外科の手術件数が年間7万件を超えている[2]。また診療科別にみると、心臓血管外科における血液製剤の使用割合は、赤血球製剤と血小板製剤では血液内科に次いで多く、新鮮凍結血漿に関しては最も高い割合を占めている[3]。心臓大血管手術において輸血量を減らすことは重要な課題である。

## **これまでに実施されてきた標準治療の経緯及び内容**

心臓大血管手術の貧血を短時間で改善する標準治療は同種赤血球輸血である。

## **現在の標準治療の内容及び治療成績**

既知のウイルスのスクリーニング検査によって同種赤血球輸血による感染症発生率が低下しているものの、未知のウイルスが新たな感染症を引き起こす可能性がある。また同種赤血球輸血自体が心臓大血管手術後の創部感染[4]や肺炎[5]、腎障害[6]と関連があることが報告されている。さらに、冠動脈バイパス術を受けた患者においては同種赤血球輸血によって死亡のリスクが増加するという報告もある（risk ratio = 1.7; 95% confidence interval = 1.4 to 2.0）[7]。

## **当該臨床試験の必要性につながる、現在の標準治療の課題、不明点など**

同種赤血球輸血に伴う問題を回避する一つの手段として自己血輸血がある。自己血輸血には貯血式、希釈式、回収式の3種の方法があるが、心臓大血管手術においては回収式が頻用される。回収式自己血輸血は、専用装置を使用して術野に出血した血液を吸引して回収し、リザーバー内のフィルターで異物を除去し貯留し、ある一定量貯留した段階で血液を濃縮・洗浄し、返血バッグに貯血して患者に戻す方法である。術中出血量が比較的多い心臓大血管手術においては、回収式自己血輸血によって同種赤血球製剤の輸血量を削減することが可能である[8-11]。2023年のコクランから出版された系統的レビュー・メタアナリシスでは、人工心肺を使用する心臓手術中に回収式自己血輸血を使用することで、同種赤血球製剤を1.47単位（95%信頼区間：0.36-2.59）削減したと報告している[12]。3年間で約1100例の心臓大血管手術を実施し、そのうち78%の症例で回収式自己血輸血を実施した本邦の施設では、3年間で合計9301単位の同種赤血球輸血を削減できたと報告している[13]。海外のガイドラインでは、人工心肺を使用する心臓手術で回収式自己血輸血を行うことを推奨している[14, 15]。

しかし、回収式自己血輸血を大量投与することで、洗浄工程で完全に除去できなかった残留ヘパリンによる凝固障害が生じるという報告がある[11]。前述した、回収式自己血輸血によって同種赤血球輸血量を削減したと報告したコクランの系統的レビュー・メタアナリシスにおける研究集団では、無輸血で終わるような出血リスクが低い術式が大半を占めていた[12]。そのため、比較的大量の輸血を要する出血リスクの高い術式で、回収式自己血の残留ヘパリンが問題になりうるヘパリン拮抗後の人工心肺離脱後に、回収式自己血を出血を増加させることなく使用できるかどうかは未解決の問題である。実際に、我々が本邦の心臓大血管手術を行っている施設に対して独自にアンケートを行った結果、40％の施設で回収式自己血が作成されたにもかかわらず使用されることなく破棄されていた。このような状況を鑑みると、出血リスクの高い術式に対する回収式自己血輸血の出血量に与える影響を検証することが喫緊の課題である。

## **試験実施の妥当性**

本研究で回収式自己血が同種赤血球輸血と比較して出血量を増加させないことが証明されれば、同種赤血球製剤の使用量削減、同種赤血球製剤による副作用の回避などにつながり、血液供給システムの維持、医療経済、そして患者の利益に貢献できる可能性がある。

# **目的**

本試験では、出血リスクの高い人工心肺を用いた心臓大血管手術で、人工心肺離脱後の貧血に対して回収式自己血を輸血することは、回収式自己血を使用せずに同種赤血球製剤のみを輸血する輸血療法と比較して、ICU入室後出血量が多くならないという仮説を、単盲検ランダム化比較非劣性試験で評価する

# **試験デザイン**

1. デザイン

- 施設：単機関
- 対照の種類：標準治療対照
- デザインの特徴：並行群間比較
- 無作為化：■　有（層別ブロック法）
- 盲検化：単盲検（被験者盲検）
- 試験の種類：非劣性検証試験

1. 侵襲の有無 有（軽微な侵襲を超える）
2. 介入の有無 有
3. 試料の利用 新たに試料を取得
4. 情報の利用 新たに情報を取得、かつ既存情報も利用

# **評価項目**

## **主要評価項目**

ICU入室後12時間のドレーン排液総量：12時間以内に再開胸止血術あるいは再開胸血腫除去術に至った場合、手術中の出血量をドレーン排液総量に追加して計算する。

## **副次評価項目**

1. 全身麻酔開始時からICU入室後12時間までの同種赤血球輸血量
2. ICU入室後48時間までの再開胸止血術あるいは再開胸血腫除去術の発生割合
3. ICU入室後12時間までのドレーン排液総量が1000mLを超える割合

### **安全性評価項目**

1. 以下のいずれかの臓器の感染症の発生割合（術後30日まで）：手術部位（SSI）、縦隔炎、尿路、呼吸器、中枢神経系、消化管、皮膚軟部組織など：CDCの診断基準[16]に従って定義される
2. ICU入室時の肉眼的血尿の有無の割合
3. 手術後7日以内の急性腎障害の発症割合

※2および3は回収式自己血輸血に伴う機械的溶血の指標として観察する

### **探索的評価項目**

1. ICU入室時の止血凝固機能：血液粘弾性検査結果（CTH (Heparinase Clot Time)、CT (Clot Time)、CTR (Clot Time Ratio)、CS (Clot Stiffness)、PCS (Platelet Contribution to Clot Stiffness)、FCS (Fibrinogen Contribution to Clot Stiffness)）、PT-INR、APTT、血小板数
2. 全身麻酔開始時からICU入室後12時間までの新鮮凍結血漿（クリオプレシピテートを含まない）、クリオプレシピテート、濃厚血小板輸血量
3. 手術時間
4. 人工心肺時間
5. 術後人工呼吸時間
6. ICU滞在時間
7. 死亡割合（全死亡および心血管死）
8. 回収式自己血（人工心肺残血回収後、初回に専用バッグに返血された血液）のヘパリン濃度
9. プロタミン投与量（手術中およびICU入室12時間）

※心血管死は以下の原因による死亡と定義する。

1. 心突然死
2. 心不全
3. 心筋梗塞
4. 脳卒中
5. 心血管疾患に関するその他の原因（例：動脈瘤、肺塞栓症）

# **試験製剤情報**

## **被験製剤**

### **名称・含量等**

回収式自己血輸血（Cell Saver Elite、Hemonetics）

### **包装・表示等**

自己血回収装置に付属する専用バッグに含まれる

### **品質管理**

回収処理終了後4時間以内に投与する。

### **回収から返血までの流れ**

1. 取扱説明書に従い、ディスポーザブルなどを自己血回収装置に装着する。
2. 無菌的にアスピレーションラインの袋を開け、滅菌紙に包まれた回路を術野へ渡す。
3. アスピレーションラインを術野から受け取り、無菌的にリザーバーへ接続する。
4. リザーバーの吸引圧を設定する。溶血を避けるため吸引圧は-150mmHgまでとするが、急速な出血時には吸引圧を一時的に250mmHgまで上昇させる。
5. 生理食塩水500mLに対してヘパリン15000単位を混注したヘパリン加生理食塩水でリザーバーをプライミングする。
6. 出血量に応じてアスピレーションラインのヘパリン加生理食塩液滴下量を調整する。 おおよそ回収血100mLに対し15mLで滴下する。
7. 術野の出血を自己血回収装置に吸引する。ただし、人工心肺中（ヘパリンを投与して活性化凝固時間が200秒以上を超えてからプロタミンを投与するまでの間）は人工心肺回路内に吸引する。
8. 取扱説明書に従って、回収、濃縮、洗浄処理を行う。
9. 洗浄後に専用バッグに返血し、輸血フィルターを用いて患者に投与する。

### **Cell Saver Eliteの設定**

ボウルサイズ：225mL

吸引圧：”スマートサクション”モード

脂肪除去：オフ

自動洗浄：オン

ボウルの濃縮が完了する前に血液を処理する：尋ねる

ポンプ流量制御：オン

濃縮ポンプ流量：500mL/h

最低洗浄量：1000mL

洗浄ポンプ流量：450mL/h

スマートエンプティ：ON

高速モード濃縮ポンプ流量：800mL/h

高速モード洗浄ポンプ流量：800mL/h

## **対照製剤**

### **名称・含量等**

同種赤血球輸血

### **包装・表示等**

回収式自己血輸血の専用バッグと外観上異なるバッグに含まれる

### **品質管理**

冷蔵で保存

# **対象**

本試験では、仮登録・本登録の2段階で適格性を判定する。

## **仮登録の選択・除外基準**

選択基準をすべて満たし、いずれの除外基準にも抵触しない患者を仮登録する。

## **選択基準**

1. 本人から同意の得られた患者
2. 同意取得時の年齢が40歳以上の患者
3. 人工心肺を使用し、胸骨正中切開アプローチによる心臓大血管手術を待機的に受ける（手術日の前日24時までに手術申し込みがあった）患者
4. 以下のいずれかに該当する患者（出血リスクの高い症例に該当する）
5. 過去に胸骨正中切開アプローチによる心臓大血管手術歴がある再開胸手術
6. 大動脈基部手術（ロス手術、弁輪拡大を伴う大動脈弁置換術、大動脈基部置換術）、上行大動脈置換術、弓部大動脈置換術のいずれかを行う患者
7. 三尖弁輪形成術を除く2弁以上の弁膜症手術を行う患者
8. 冠動脈バイパス手術に三尖弁輪形成術を除く弁膜症手術を合わせて行う患者

## **除外基準**

1. 下行大動脈置換術、胸腹部大動脈置換術、心臓移植術、補助人工心臓移植術、肺動脈弁置換術のいずれかを受ける患者
2. 出血リスクが極めて高いと考えられる患者：胸骨正中切開による心臓大血管手術を過去に3回以上受けた患者と定義する。その他、出血リスクが極めて高いと判断される症例については、担当外科医および麻酔科医の協議により最終的に除外の可否を決定する。
3. 術前の抗血小板薬または抗凝固薬の休薬期間を遵守していない患者（表1参照）
4. 活動性の細菌あるいはウイルス感染症を合併している患者
5. 妊娠中または授乳中の患者
6. RhD抗原陰性患者
7. 臨床的に意義のある不規則抗体を保有する、あるいは過去に臨床的意義のある不規則抗体の保有歴があり、該当する不規則抗体陰性血液製剤の入手が限定される場合（臨床的意義の有無は「赤血球型検査（赤血球系検査）ガイドライン（改訂4版）」[17]を参考にして判断する）
8. その他の理由により、研究責任者または分担者が本試験への参加を不適当と判断した患者

表1：術前の抗血小板薬または抗凝固薬の休薬期間

| 一般名 | 休薬期間 |
| --- | --- |
| 抗血小板薬 | |
| アスピリン | 7日 |
| アスピリン+タケキャブ | 7日 |
| アスピリン＋ランソプラゾール | 7日 |
| アスピリン＋クロピドグレル | 7日 |
| クロピドグレル | 5日 |
| プラスグレル | 7日 |
| チクロピジン | 7日 |
| チカグレロル | 3日 |
| シロスタゾール | 3日 |
| 抗凝固薬 | |
| ワルファリン | 3日 |
| ダビガトラン | 3日 |
| リバーロキサバン | 3日 |
| アピキサバン | 3日 |
| エドキサバン | 3日 |

※休薬期間がx日の薬剤については、最終内服時刻からx日（すなわちx × 24時間）以上が経過していることをもって、所定の休薬期間を満たしているものと判断する。

## **設定根拠**

### **選択基準の設定根拠**

1)：介入試験のため、本人による文書同意を必須とした。

2)-4)：本試験の対象患者を特定するために設定した。

### **除外基準の設定根拠**

1)- 3) 5)：本試験の評価に影響を及ぼすことを考慮して設定した。

4)- 8)：被験者の安全性を考慮して設定した。

## **本登録の選択・除外基準**

　仮登録された症例は、以下の選択基準を満たし、除外基準に抵触していないことを確認した上で本登録する。

## **選択基準**

人工心肺中に同種赤血球輸血を実施した患者

## **除外基準**

1. 仮登録後から手術日までの間に、抗血小板薬または抗凝固薬の休薬期間（表1参照）が遵守されていなかった場合
2. 仮登録後から手術日までの間に、あるいは手術中に手術術式が変更し、仮登録の選択基準から抵触するか、仮登録のいずれかの除外基準を満たした場合

## **設定根拠**

### **選択基準の設定根拠**

設定した基準を満たさない場合、無輸血手術になる可能性が高く、本試験が対象とする出血リスクの高い集団から外れることになる。無輸血手術とは、自己血のみで貧血を是正でき、同種血製剤を投与する必要性がない手術を指し、出血量が少ない場合でないとこれを達成できない。

### **除外基準の設定根拠**

本試験の評価に影響を及ぼすことを考慮して設定した。

# **試験の実施手順**

## **インフォームド・コンセント**

### **試験参加の任意性**

本臨床試験は、ヘルシンキ宣言に基づく倫理原則及び「人を対象とする生命科学・医学系研究に関する倫理指針」に従って行われる。本試験への参加もしくは不参加は被験者個人の自由であり、被験者の基本的人権に配慮する。本試験への参加の有無にかかわらず、被験者に対する診療は最大限適切なものが行われる。説明文書を用いて、被験者に対して十分に説明を行い、被験者自身から文書による同意を得る。

### **インフォームド・コンセントを受ける手続き**

研究責任者または研究分担者は、患者が本試験に参加するかどうかを意思決定するために、説明文書を用いて説明し、試験参加への検討をするための十分な時間を設けたうえで、自由意思による試験参加への同意を本人から文書により受ける。

文書による同意を得る際には、患者は説明文書の内容を十分理解したうえで同意し、同意文書に同意日を記入するとともに署名する。説明を行った研究責任者又は分担者が、同意文書に説明日を記入し、署名する。

### **再同意**

試験継続についての被験者の意思決定に影響を与えると判断される試験実施計画書の改訂が行われた場合は、倫理審査委員会に改訂した説明文書・同意文書の承認を得た後、その改訂内容を被験者に説明し、文書による再同意を得る。

### **同意の撤回**

一旦被験者より同意を得た場合でも、被験者は不利益を受けることなく、原則としていつでも同意を撤回することができる。ただし、すでに研究結果を発表している場合には同意撤回に応じることができない。その他、事前に想定できなかった理由により同意撤回の措置を講じることが困難な場合は、倫理審査委員会に諮り、同意撤回の措置を講じないことについて許可を得る。

同意撤回以降は、その後の本試験に関する情報等の追加収集は行わない。また、未利用の試料と情報については以後の利用を不可とする。

説明文書に同意撤回できる旨と、撤回できない場合があること及びその理由、申出先・担当者を記載し、説明する。試験参加に同意した被験者から、同意撤回の意思表示があった場合、その旨を同意撤回書若しくはカルテの記録に残し、撤回意思に従った措置を講じたことを被験者に説明する。同意撤回できない場合にはその旨を説明して理解を得るよう努める。

## **被験者の仮登録**

研究責任（分担）者は、患者本人から同意を取得する。研究責任（分担）者は、仮登録の選択基準に合致し、除外基準に抵触しない者を仮登録適格とする。研究責任（分担）者は、被験者識別番号を入手する。仮登録不適格の場合は、この時点で終了となる。終了後は、通常の診療を行う。仮登録の適格性基準を満たさなかった理由は、スクリーニング記録に記載する。

＜目標症例数の到達＞

本治験は目標症例数として、本登録症例数142 を設定している。142例目の症例が仮登録された際に一旦同意取得を中断する。その後、不足症例を登録する。

## **被験者の本登録**

本登録の選択基準を満たし、除外基準に抵触しないものを本登録適格とする。本登録不適格の症例は、この時点で終了となる。終了後は、通常の診療を行う。手術終了後に、本登録不適格例となったことを伝える。

## **割り付けと盲検化**

研究責任者または研究分担者は、本登録後、Web登録システムにアクセスし、登録に必要な情報を入力して送信する。被験者は回収式自己血群あるいは同種赤血球輸血群のいずれかに1:1の割合で無作為に割り付けられる。割り付けは層別ブロック法を用いる。

本試験では、脳分離体外循環を伴う弓部大動脈置換術の実施有無と年齢（40~64歳、65歳~）を割り付け調整因子とする。

本試験は、被験者、評価者、執刀医と手術助手、術後管理に関わる医師に対して、試験期間を通じて盲検性が保持されるよう実施する（該当手術に関わる麻酔科医のみ非盲検とする）。術後管理に関わる医師に対する盲検解除については、術後管理医師がコントロールできない出血によって盲検解除が必要と判断した場合に行う。

## **試験製剤の投与**

本試験のスケジュールは以下のとおりである。手術当日までに同意取得・仮登録し、人工心肺中に本登録/割り付け後、人工心肺離脱時（プロタミン投与を開始する時）より、以下の投与基準に従い、試験製剤の投与を行う。

＜試験製剤の投与基準＞

試験製剤は血液検査でヘモグロビン10.0g/dL未満の貧血を確認した際、あるいは貧血が想定される際に投与する。


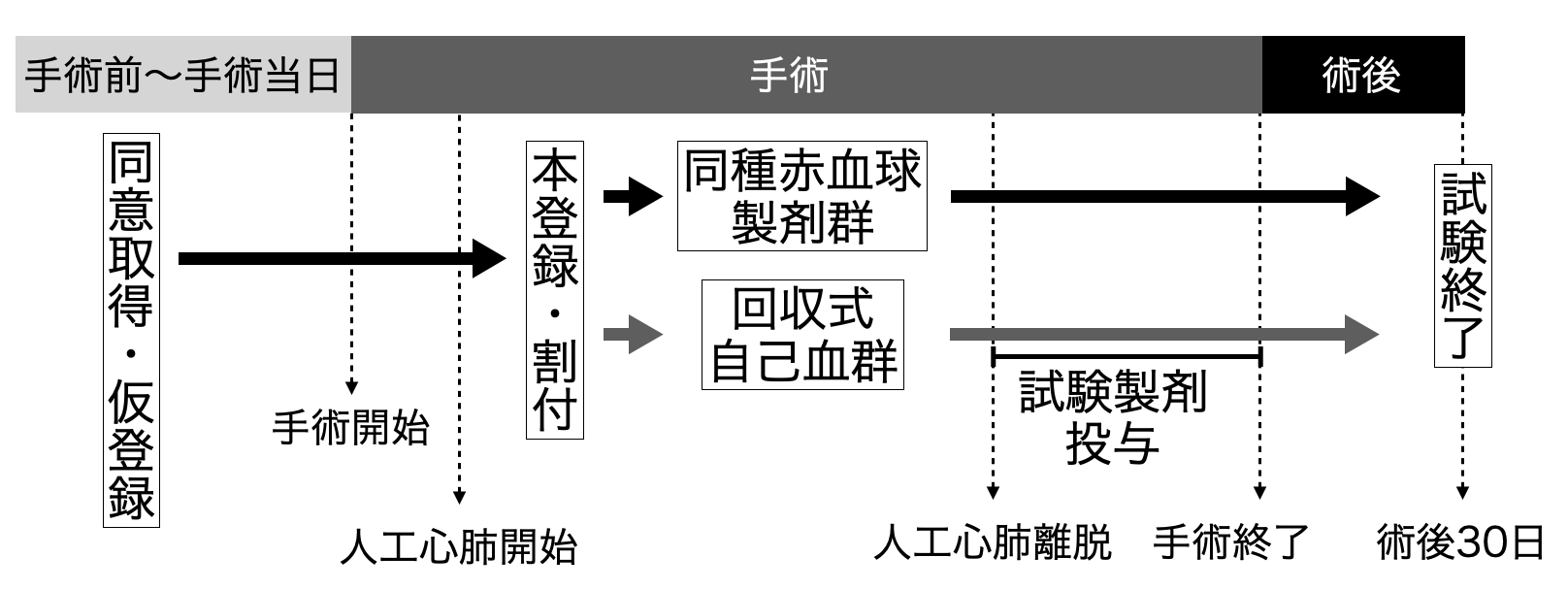


### **回収式自己血群（被験製剤群）**

人工心肺離脱時から手術室退室時までの貧血に対して、回収式自己血を同種赤血球製剤に優先して使用する。人工心肺離脱後の人工心肺回路内に残った血液（人工心肺残血）は、送血管と脱血管を抜去後速やかに回収式自己血装置内に回収する。回収式自己血を使いきってもなお貧血が是正されていない場合、あるいは是正されていないことが想定される場合は同種赤血球製剤を輸血する。同種赤血球製剤を輸血している際に回収式自己血が生成された場合は、同種赤血球製剤の輸血を中断し、回収式自己血の輸血を開始する。輸血の必要性が生じた際に回収式自己血が生成されていなければ同種赤血球製剤を輸血する。人工心肺残血回収後、初回に専用バッグに返血された血液2mLを採取し、ヘパリン濃度を測定するために委託先に送る。

ICU帰室以降の貧血に対しては同種赤血球製剤を使用する。

### **同種赤血球製剤群（対照製剤群）**

人工心肺離脱時から手術室退室時までの貧血に対して、回収式自己血を使用せず、同種赤血球製剤のみ輸血する。人工心肺離脱後の人工心肺回路内に残った血液（人工心肺残血）は、送血管と脱血管を抜去後速やかに回収式自己血装置内に回収するが、専用バッグに返血された自己血は輸血しない。

### **試験治療の中止基準**

- 回収式自己血群において、回収血内に患者体内の感染による膿などが混入し、回収式自己血を行うことで新たな感染症を生じる可能性が生じた場合
- 同種赤血球製剤群において、同種赤血球製剤が不足しているために回収式自己血を使用しなければ危機的な貧血を生じうると執刀医あるいは麻酔科医が判断した場合
- 手術中の予期せぬ合併症により、極めて出血リスクの高い術式へ変更した場合
- 手術中に補助循環装置が必要となった場合
- 研究責任者、研究分担者が試験継続が困難であると判断した場合

## **前治療および併用療法**

### **研究として定める前治療**

- ノイアート（人アンチトロンビンⅢ製剤）：人工心肺開始前にヘパリン300-500U/kg投与後に活性化凝固時間が400秒を超えないときに500-1500U投与する。
- ケイセントラ（静注用人プロトロンビン複合体製剤）：ワーファリンの休薬期間を遵守しているにも関わらず手術当日のPT-INRが正常上限を超えている場合に、手術開始までに500-1000U投与する。

### **併用療法**

- 新鮮凍結血漿輸血：人工心肺離脱後に開始し、手術中の新鮮凍結血漿輸血総量／赤血球輸血総量（回収式自己血輸血量＋同種赤血球輸血量）の比が1を超えないように輸血する。フィブリノゲン200mg/dL以上、かつ出血傾向を認めないときは新鮮凍結血漿輸血を実施しない。
- クリオプレシピテート：フィブリノゲン100mg/dL未満のときに、新鮮凍結血漿12単位分を投与する。フィブリノゲン100-149mg/dLのときに新鮮凍結血漿12単位分の投与を考慮する。
- トラネキサム酸：手術開始時に1gボーラス投与し、2mg/kg/hで手術終了時まで持続投与する。
- プロタミン：人工心肺離脱時に3mg/kg投与する。その後は血液粘弾性検査（Quantra）でCTR（clotting time ratio）1.2以上の場合、あるいはACTがH-ACT（ヘパリナーゼを用いてヘパリンを分解した ACT）を上回る場合に追加投与する。
- ボルベン（膠質輸液製剤）：手術中の総投与量を50mL/kgを上限とする。

### **後治療**

ICUに入室してから12時間の輸血療法を以下のように定める。

- 赤血球輸血：ヘモグロビン10.0g/dL未満であること、あるいは10.0g/dL未満であることが想定される場合に行う。赤血球輸血は同種赤血球輸血を行い、手術中に作成し余った回収式自己血を投与しない。12時間以内の再開胸手術中に輸血が必要となった場合には、両群とも同種赤血球製剤のみを輸血する。12時間以降の再開胸手術中の輸血に関しては、回収血自己血あるいは同種赤血球製剤か制限をしない。
- 血小板輸血：血小板数10.0×10^4^/μL未満、かつ臨床的な出血傾向があるときに実施する。
- 新鮮凍結血漿輸血：APTT>50秒あるいはPT-INR>1.3あるいはフィブリノゲン<150mg/dLで、出血傾向があるときに実施する。ただし、血液粘弾性検査（Quantra）でCTR（clotting time ratio）1.2以上の場合には、プロタミンを次項で記載のように投与し、再度計測したAPTT値を評価する。大量出血時にはICUでの新鮮凍結血漿輸血総量／同種赤血球輸血総量の比が1を目安に実施する。
- プロタミン：血液粘弾性検査（Quantra）でCTR（clotting time ratio）1.2以上の場合に追加投与する。あるいはACTがH-ACT（ヘパリナーゼを用いてヘパリンを分解した ACT）を上回る場合に追加投与する。

## **試験治療の中止基準およびその手順**

### **試験製剤投与の中止手順**

試験製剤投与の中止基準に該当する場合、研究責任(分担)者は試験製剤投与を中止する。中止日および中止理由について、カルテ等に記録を残す。試験製剤投与が中止となった被験者においても、試験終了まで予定された観察項目の収集を継続する。試験製剤投与によると思われる有害事象により試験製剤投与の継続が困難と判断された場合には、有害事象発生の対応手順に従い、適切に対処する。

## **被験者の試験参加の中止基準およびその手順**

### **被験者の試験参加の中止基準**

下記の理由で、被験者が試験製剤投与の中止だけでなく、すべての試験スケジュールが出来なくなった場合、研究責任(分担)者は被験者の試験参加を中止する。

1. 被験者が試験参加の中止を希望した場合
2. 同意取得後に手術が中止になった場合
3. 安全性の観点から、研究責任者が被験者の試験参加の中止が必要と判断した場合
4. 試験全体が中止となった場合
5. その他、研究責任(分担)者が試験参加の継続が困難であると判断した場合

### **被験者の試験参加の中止手順**

被験者の試験参加の中止基準に該当する場合、研究責任者は試験を中止し、中止日および試験を中止した理由をカルテ等に記録する。被験者が試験の中止を希望した場合、それ以降のデータ収集は原則行わない。

# **観察・検査項目とスケジュール**

## **Visit 1. 同意取得・適格性確認・仮登録**

手術30日前から手術当日までに、通常診療で得られている以下の情報を収集して適格性を確認し、同意取得を行う。

| １） 被験者基本情報 | |
| --- | --- |
| 被験者情報 | 同意取得日、生年月日、年齢、性別 |
| ２）対象疾患情報 | |
| 疾患情報 | 診断名、予定術式、既往症、併存疾患 |
| ３）検査情報**^※^** | |
| 身体所見 | 身長、体重、体表面積 |
| 末梢血算・凝固検査・血液型関連検査 | ヘモグロビン値、血小板数、PT-INR、APTT、血液型（ABO、RhD）、不規則抗体の有無 |
| ４）内服薬物情報 | |
| 内服薬剤 | ステロイド・免疫抑制薬・抗血小板薬および抗凝固薬の内服有無、休薬期間 |

- 同意取得前90日以内の検査結果を用いることとする。該当する結果が複数存在する場合は同意取得日に最も近いタイミングで実施された結果を研究として採用する。

## **Visit 2.** **本登録・割り付け（ベースライン）**

手術当日の人工心肺離脱時までに、以下の項目をベースライン情報として収集する。

| １）検査情報**^※2^** | |
| --- | --- |
| 身体所見 | 身長、体重、体表面積 |
| 末梢血算・生化学・凝固検査 | 白血球数、ヘモグロビン、血小板数、総蛋白、アルブミン、総ビリルビン、直接ビリルビン、AST、ALT、クレアチニン、LDH、CK、CK-MB、ナトリウム、カリウム、クロール、カルシウム、CRP、PT-INR、APTT、フィブリノゲン、D-dimer、FDP、ATⅢ活性 |
| ２）内服薬物情報 | |
| 内服薬剤 | 抗血小板薬および抗凝固薬の休薬期間 |
| ３）手術情報 | |
| 治療情報 | 人工心肺中の同種血輸血の実施有無 |

※2全身麻酔開始前までに情報を収集する。手術日の前 14日以内に通常診療で得られている結果がある場合は、その結果を用いることとする。

## **Visit 3. 試験治療中**

手術当日の人工心肺離脱時以降（許容範囲：+3日）に以下の項目の情報を収集する。情報は麻酔記録：ORSYSと、電子カルテ：Megaoakから収集する。

| １）試験治療情報 | |
| --- | --- |
| 治療情報 | ・全身麻酔開始時〜人工心肺離脱時、人工心肺離脱時〜手術室退室時の回収式自己血投与量、同種赤血球製剤投与量  ・術中出血量  ・回収血のヘパリン濃度（回収式自己血群のみ）^※3^ |
| ２）検査情報 |  |
| バイタルサイン | 血圧、心拍数、経皮的酸素飽和度、中心静脈圧、肺動脈圧、心拍出量（手術終了時） |
| 身体所見 | 肉眼的血尿の有無（ICU入室時） |
| 血液ガス | ヘモグロビン（全身麻酔導入後、人工心肺開始前、人工心肺中、人工心肺離脱後でプロタミン投与後、さらに適宜必要に応じて実施された検査情報を収集する） |
| 末梢血算・凝固検査 | ヘモグロビン、血小板、PT-INR、APTT、フィブリノゲン、血液粘弾性検査（人工心肺離脱前、人工心肺離脱後でプロタミン投与後、ICU帰室時、適宜必要に応じて実施された検査情報を収集する） |
| 生化学検査 | 総蛋白、アルブミン、総ビリルビン、直接ビリルビン、AST、ALT、クレアチニン、LDH、CK、CK-MB、ナトリウム、カリウム、クロール、カルシウム、CRP （ICU帰室時と、適宜必要に応じて実施された検査情報を収集する） |
| ２）併用治療情報 | |
| 併用療法 | ・全身麻酔開始時〜人工心肺離脱時、人工心肺離脱時〜手術室退室時の新鮮凍結血漿・血小板濃厚液・クリオプレシピテートの投与量  ・膠質輸液、トラネキサム酸、ヘパリン（自己血回収装置に仕様したヘパリンは除く）、プロタミンの手術中総投与量 |
| ３）手術情報 | |
| 手術情報 | 確定診断、確定術式、手術時間、人工心肺時間、心停止時間、人工心肺中最低体温 |
| ４）有害事象 | |
| 有害事象 | 発現日、事象名、既知・未知、重篤度、重症度、因果関係、処置、転帰、転帰確認日、その他詳細 |

※3研究目的に測定

## **Visit 4. 試験終了時**

試験治療開始後30日（許容範囲：前後5日）に、下記の観察・検査を行う。情報は、重症患者管理システム：アクシスと電子カルテ：Megaoakから収集する。

| １）試験治療情報 | |
| --- | --- |
| 治療情報 | ICU入室後12時間のドレーン排液総量、ドレーン抜去までの総排液量、ICU入室後24時間輸血量（赤血球製剤、新鮮凍結血漿、クリオプレシピテート、血小板製剤）、人工呼吸時間、ICU滞在時間、術後入院期間、退院日 |
| 治療中止／中断の有無 | 有無、中止／中断有の場合：中止／中断日、中止／中断理由、中止／中断後の処置 |
| ２）検査情報 | |
| 末梢血算・生化学・凝固検査 | 白血球数、ヘモグロビン、血小板数、総蛋白、アルブミン、総ビリルビン、直接ビリルビン、AST、ALT、クレアチニン、LDH、CK、CK-MB、ナトリウム、カリウム、クロール、カルシウム、CRP、PT-INR、APTT、フィブリノゲン（手術翌日に実施された情報を取得する） |
| ３）有害事象報 | |
| 有害事象 | 発現日、事象名、既知・未知、重篤度、重症度、因果関係、処置、転帰、転帰確認日、再入院の有無・入院日・退院日、その他詳細 |
| ４）転帰情報 | |
| 生存確認 | 転帰：生存、死亡、追跡不能  最終生存確認日もしくは死亡日、死亡の場合：死因  追跡不能の場合：追跡不能理由 |
| 参加中止 | 有無　中止有の場合：中止日、中止理由 |

## **試験早期中止時　（中止決定日＋3）**

試験の早期中止が決定した日から３日以内に、下記の観察・検査を行う。

| １）試験治療情報 | |
| --- | --- |
| 治療中止／中断の有無 | 有無、中止／中断有の場合：中止／中断日、中止／中断理由、中止／中断後の処置 |
| ２）検査情報^※4^ | |
| 末梢血算・生化学・凝固検査 | 白血球数、ヘモグロビン、血小板数、総蛋白、アルブミン、総ビリルビン、直接ビリルビン、AST、ALT、クレアチニン、LDH、CK、CK-MB、ナトリウム、カリウム、クロール、カルシウム、CRP、PT-INR、APTT、フィブリノゲン |
| ３）有害事象情報 | |
| 有害事象 | 発現日、事象名、既知・未知、重篤度、重症度、因果関係、処置、転帰、転帰確認日、再入院の有無・入院日・退院日、その他詳細 |
| ４）転帰情報 | |
| 生存確認 | 転帰：生存、死亡、追跡不能  最終生存確認日もしくは死亡日、死亡の場合：死因  追跡不能の場合：追跡不能理由 |
| 参加中止 | 有の場合：中止日、中止理由 |

※4中止決定日の前1日以内に行われた他Visitの検査結果及び通常診療での検査結果がある場合には、その結果を用いることとする。**観察・検査スケジュール表**

| 調査時期  調査項目 | 試験期間 | | | | 早期中止 |
| --- | --- | --- | --- | --- | --- |
|  | Visit 1 | Visit 2 | Visit 3 | Visit 4 |  |
|  | **同意取得・**  **適格性確認・仮登録** | **本登録・割り付け（試験治療開始前）** | **試験治療中** | **試験終了** |  |
| 規定日 | -30日～ | 0日 | 0日 | 30日 | ― |
| 許容範囲 | ― | ― | ＋3日 | ±5日 | ＋3日 |
| **同意取得** | ◎ |  |  |  |  |
| **被験者基本情報** | ○ |  |  |  |  |
| **対象疾患情報** | ○ |  |  |  |  |
| **試験治療情報** |  |  | ○ | ○ | ○ |
| **内服薬剤情報** | ○ | ○ |  |  |  |
| **身体所見** | ○^※1^ | ○^※2^ | ○ |  |  |
| **バイタルサイン** |  |  | ○ |  |  |
| **血液検査（末梢血算・生化学、血液型関連、止血・凝固、血液粘弾性検査）** | ○^※1^ | ○^※2^ | ○ | ○^※3^ | ○^※4^ |
| **血液ガス** |  |  | ○ |  |  |
| **回収血ヘパリン濃度**  **（回収式自己血群のみ）** |  |  | ● |  |  |
| **併用治療情報** |  |  | ○ |  |  |
| **手術情報** |  | ○ | ○ |  |  |
| **有害事象** |  |  |  |  |  |
| **転帰情報** |  |  |  | ○ | ○ |

　➡：期間を通じて調査する　◎：必須項目　○：通常診療の検査項目　●：研究目的の検査

※1同意取得前90日以内に通常診療で得られている結果を研究として使用する。

※2全身麻酔開始前までに情報を収集する。手術日の前14日以内に通常診療で得られている結果がある場合は、その結果を用いることとする。

※3手術翌日に実施された情報を取得する。

※4可能な限り実施する。中止決定日の前1日以内に行われた他Visitの検査結果及び通常診療での検査結果がある場合には、その結果を用いることとする。

## **観察・検査項目の定義**

### **疾患の定義**

|  | 定義 |
| --- | --- |
| 心筋梗塞 | 左記疾患として循環器医により確定診断を受けたもの。 |
| 脳卒中 | 左記疾患として脳神経外科医または、脳神経内科医により確定診断を受けたもの |
| 心不全 | 左記疾患として入院歴のあるもの、またはNYHAII以上、心エコーによりEF50％未満。 |
| 既知の凝固・線溶障害 | 特発性血小板減少症、血栓性血小板減少症、再生不良性貧血、燐脂質抗体、プロテインC欠損症、他先天性、後天性の凝固、線溶系障害と確定診断を受けたもの |

# **有害事象の評価・報告**

## **有害事象の定義**

有害事象とは、試験治療を受けた被験者に生じたあらゆる好ましくない医療上の出来事をいう。必ずしも試験治療との因果関係は問わない。また、副作用は、試験製剤との因果関係が合理的に否定できない有害事象とする。

## **有害事象発現時の対応**

有害事象が発生した場合には、速やかに被験者に対し適切かつ最善の対応を行う。試験中に発生した有害事象については、①有害事象名、②発現日、③既知・未知、④重篤性の有無、⑤重症度、⑥因果関係、⑦処置等の対応などについて、カルテ等に速やかに記載する。全ての有害事象に関して、重篤の基準に該当するかを判断する。重篤な有害事象と判断された場合、9.4.2記載されている手順に従い、報告する。また因果関係があると判断された有害事象に関しては、原則として回復もしくは軽快するまで追跡調査を行い、有害事象の転帰および転帰日について記録する

## **有害事象の評価**

### **重篤な有害事象の定義**

重篤な有害事象とは、有害事象のうち、下記に該当するものを指す。

　　１．死亡

　　２．死亡につながるおそれのあるもの

　　３．治療のために入院または入院期間の延長が必要となるもの

　　４．障害

　　５．障害につながるおそれのあるもの

６．その他、１～５に準じて重篤である

７．後世代における先天性の疾病または異常をきたすおそれのあるもの

なお、3.の「入院」について、同意取得前より予定していた治療または検査を、試験参加中に実施することのみを目的とした入院（予定手術や検査等）、管理上または社会的な理由による入院（点滴実施日、実施医療機関が自宅から遠い等の理由で日帰り治療が入院となる場合など）、有害事象に伴う治療の目的以外の入院（検査入院、健康診断等）は、重篤な有害事象として取扱わない。

### **有害事象の重症度**

本試験の有害事象の重症度を以下に分類する。

| 重症度 | 定義 |
| --- | --- |
| 軽度 | 兆候または症状を自覚するが、被験者の通常の活動を妨げない、あるいは一過性で治療せずに回復し、後遺症を残さないもの |
| 中等度 | 被験者の通常の活動を妨げるまたは処置を必要とするもの |
| 重度 | 被験者の通常の活動へ重大な影響を及ぼすと共に、外科的治療等を必要とし、死亡または日常生活に支障をきたす程度の永続的な機能不全に陥る恐れのあるもの |

### **試験製剤との因果関係**

研究責任(分担)者は、有害事象について試験製剤との因果関係について評価する必要がある。試験製剤との因果関係を「否定できない」と「否定できる」の2区分で判断する。研究責任者が、試験製剤との因果関係がないと合理的に判断できない場合には、「否定できない」として評価する。

## **有害事象の報告**

### **有害事象の報告期間**

報告対象となる有害事象は、試験製剤投与開始時から30日後もしくは本試験実施計画書に規定されている最終来院日の遅い時点までとする。試験製剤との因果関係が否定できない重篤な有害事象は、上記の報告期間以外であっても、試験期間終了時まで報告する。

### **重篤な有害事象の報告手順**

研究責任者並びにその他の試験の実施に携わる全ての担当者は、本試験に参加している被験者において重篤な有害事象を知り得た場合、以下の手順に従って対応を実施するものとする。

＜対応手順＞

1. 重篤な有害事象の発生を知り得た場合には、速やかに研究責任者へ報告する。
2. 研究責任者は、速やかに倫理審査委員会および研究統括管理責任者に報告する。
3. 研究責任者は、当該有害事象の発生に関わる情報を研究機関の長に報告するとともに、計画書及び研究機関の手順書等に従い、必要な対応を講じる。また、速やかに試験に携わる研究者等へも情報共有する。
4. 研究責任者は倫理審査委員会から通知を受けた際は、研究機関の長へ報告する。また、倫理審査委員会から述べられた意見に基づき具体的な対応が必要な場合には、その対応の内容を含めて研究機関の長に報告する。
5. 当該有害事象が、本研究の実施において予測できないものであり、かつ、本試験との因果関係が否定できない事象である場合には、研究責任者は、研究機関の長に報告した上で、速やかに上記に定めた手順による対応の状況及び結果を厚生労働大臣に報告（報告書式：倫理指針様式3）し、ホームページで公表する。

## **予測される有害事象等**

本試験製剤の投与で予測される有害事象は下記の通りである。

### **共通**

- 発熱
- 低体温
- 高カリウム血症
- 希釈性凝固障害
- アシドーシス

### **同種赤血球製剤に特異的なもの**

- 溶血性副作用
- 輸血関連呼吸困難
- アレルギー反応
- 輸血関連肺障害
- 輸血後関連循環過負荷
- 輸血後紫斑病
- 輸血後移植片宿主病
- 輸血後ウイルス性、細菌性、原虫性、その他の感染症
- 輸血後鉄過剰症
- 低カルシウム血症

### **回収式自己血輸血に特異的なもの**

- 細菌性感染症
- 溶血性副作用

　本試験では上記以外に手術に関連する合併症が生じうる。この情報も有害事象として収集する。

# **試験期間**

予定試験期間：研究許可日～2030年3月31日

症例登録期間：研究許可日～2026年9月30日

症例観察期間：研究許可日～2026年11月4日

# **目標症例数**

目標症例数：本登録症例として142例（回収式自己血群 71例、同種赤血球製剤群 71例）

# **統計的事項**

　別紙：統計解析計画書に従う。

# **倫理的事項**

## **遵守すべき諸規則**

本試験は、ヘルシンキ宣言に基づく倫理的原則、「人を対象とする生命科学・医学系研究に関する倫理指針」、及び試験実施計画書を遵守して実施する。

## **試験実施の手続き**

研究責任者は、本試験を実施する前に倫理審査委員会の承認及び研究機関の長の実施許可を得る。

## **研究機関の長及び倫理審査委員会への報告**

１）試験実施中の報告事項

研究者等は以下の①～③に該当する事実や情報を得た場合、研究責任者へ報告する。②③に該当する場合、研究者等は速やかに研究機関の長に報告し、必要に応じて試験を停止し、もしくは中止し、又は試験実施計画書を変更する。（倫理指針第11の1,2(2), 2(3)）

①　試験の倫理的妥当性、科学的合理性を損なう又はそのおそれのある事実や情報

②　試験の実施の適正性、試験結果の信頼を損なう又はそのおそれがある事実や情報

③　試験に関連する情報の漏洩等、被験者等の人権を尊重する観点又は試験の実施上の観点から重大な懸念が生じた場合

２）定期報告

研究責任者は、年に1回の頻度で試験の進捗状況及び研究の実施に伴う有害事象の発生状況を、倫理審査委員会及び研究機関の長に報告する。

３）研究終了時の報告（中止の場合を含む）

研究責任者は、試験終了時に遅滞なく倫理審査委員会及び研究機関の長に報告する。

## **試験参加に伴う被験者の利益と不利益の総合的評価**

### **試験参加に伴う被験者の利益および試験がもたらす利益**

　本試験において、心臓大血管手術において回収式自己血がICU入室後出血を増加させないということが明らかになればとなれば、これまで廃棄されていた回収式自己血を有用に活用でき、同種赤血球製剤の輸血量を減少できる可能性がある。しかし、本試験期間中に、被験者の治療に際して何らかの利益を与えることができる可能性は低いと考えられる。

### **試験参加に伴う被験者の負担**

本研究は研究用にヘパリンの濃度を測定する採血を行うが、生成された回収式自己血から2mLを採取するため、穿刺リスクはなく、また貧血の進行等のリスクは極めて低い。

### **試験参加に伴う被験者への予測されるリスク**

試験製剤投与により、9.5に記載されている副作用等が出現する可能性がある。また、研究用の血液採取により、貧血の進行等のリスクが存在するが極めて低い。被験者の個人情報は、特定の個人を直ちに識別することができないよう加工し管理を行うため、個人情報の外部漏えいに基づく不利益が生じるリスクは低いと思われる。

### **被験者のリスクを最小化するための対策**

　患者の個人情報は加工するため、外部漏えいに基づく不利益が生じるリスクは低いと思われる。

### **総合評価**

　本試験がもたらす総体としての利益は、本試験に伴う不利益を正当化するものであると判断する。

### **試験参加に伴う被験者への謝礼等**

本試験では、試験参加に伴う被験者への謝礼は支払わない。

## **被験者及びその関係者からの相談等への対応**

　説明文書に相談窓口として試験事務局の連絡先（住所、電話番号等）を記載し、被験者等及びその関係者から本試験に関する相談等に対応する。

## **説明文書・同意文書の作成と改訂**

### **説明文書の作成および研究機関の長の許可**

研究責任者は、説明文書・同意文書を作成する。また、作成した説明文書・同意文書は試験開始前に研究機関の長に提出し許可を得る。

### **説明文書への記載事項**

説明文書には、「人を対象とする生命科学・医学系研究に関する倫理指針」に定められた以下の事項を記載する。

1. 研究の名称及び当該研究の実施について研究機関の長の許可を受けている旨
2. 当該研究対象者に係る研究協力機関の名称、既存試料・情報の提供のみを行う者の氏名及び所属する機関の名称並びに全ての研究責任者の氏名及び研究機関の名称
3. 研究の目的及び意義
4. 研究の方法（研究対象者から取得された試料・情報の利用目的を含む。）及び期間
5. 研究対象者として選定された理由
6. 研究対象者に生じる負担並びに予測されるリスク及び利益
7. 研究が実施又は継続されることに同意した場合であっても随時これを撤回できる旨（研究対象者等からの撤回の内容に従った措置を講じることが困難となる場合があるときは、その旨及びその理由）
8. 研究が実施又は継続されることに同意しないこと又は同意を撤回することによって研究対象者等が不利益な取扱いを受けない旨
9. 研究に関する情報公開の方法
10. 研究対象者等の求めに応じて、他の研究対象者等の個人情報等の保護及び当該研究の独創性の確保に支障がない範囲内で研究実施計画書及び研究の方法に関する資料を入手又は閲覧できる旨並びにその入手又は閲覧の方法
11. 個人情報等の取扱い（匿名化する場合にはその方法、匿名加工情報又は非識別加工

情報を作成する場合にはその旨を含む。）

1. 試料・情報の保管及び廃棄の方法
2. 研究の資金源等その他の研究機関の研究に係る利益相反及び個人の収益その他の研究者等の研究に係る利益相反に関する状況
3. 研究により得られた結果等の取り扱い
4. 研究対象者等及びその関係者からの相談等への対応
5. 研究対象者等に経済的負担又は謝礼がある場合には、その旨及びその内容
6. 通常の診療を超える医療行為を伴う研究の場合には、他の治療方法等に関する事項
7. 通常の診療を超える医療行為を伴う研究の場合には、研究対象者への研究実施後における医療の提供に関する対応
8. 侵襲を伴う研究の場合には、当該研究によって生じた健康被害に対する補償の有無及びその内容
9. 研究対象者から取得された試料・情報について、研究対象者等から同意を受ける時点では特定されない将来の研究のために用いられる可能性又は他の研究機関に提供する可能性がある場合には、その旨、同意を受ける時点において想定される内容並びに実施される研究及び提供先となる研究機関に関する情報を研究対象者等が確認する方法
10. 侵襲（軽微な侵襲を除く。）を伴う研究であって介入を行うものの場合には、研究対象者の秘密が保全されることを前提として、モニタリングに従事する者及び監査に従事する者並びに倫理審査委員会が、必要な範囲内において当該研究対象者に関する試料・情報を閲覧する旨

### **説明文書・同意文書の改訂**

試験開始後に研究責任(分担)者が被験者の同意に関連する新たな知見を得て、説明文書・同意文書の改訂が必要と判断した場合には、当該情報に基づき説明文書・同意文書を改訂する。被験者の同意に関連する新たな知見とは、例えば当該治療法等に関連する新たな有害事象の情報、あるいは当該疾患に関わる新治療法等の開発に関する情報等を指す。なお、改訂の内容が被験者の試験継続についての意思決定に影響を与えると判断する場合は倫理審査委員会に改訂した説明文書・同意文書を提出し、その承認を得た後、再度被験者に説明し、文書による同意を得る。

# **個人情報の保護**

被験者の個人情報を保護するため、本試験の実施にあたり下記の対応を遵守する。

1. 被験者に関わるデータを取り扱う際は、被験者の秘密保護に十分配慮する。
2. 試験で取り扱う個人情報は、本研究に必要なものに限定し、被験者の氏名を当該試験固有の識別番号に置き換え、特定の個人を直ちに識別することができないよう加工し管理する。
3. 個人情報の加工、すみやかに解析開始までに行う。
4. 対応表は、当センター「研究等における個人情報管理に関する規程」に基づき、解析開始までに研究等個人情報管理室へ提出する。それまでは研究責任者の責任の下、医局内個人デスクの施錠可能な引き出しに保管する。

# **データの管理**

## **症例報告書の作成**

研究責任(分担)者もしくは研究協力者は、観察・調査項目に関するデータ等を記載し、症例報告書を作成する。症例報告書の記載内容は、原資料と矛盾がないことを確認する。症例報告書の内容をMicrosoft Excelに入力し、データを管理する。

## **症例報告書の変更・修正**

症例報告書の変更もしくは修正する必要がある場合、研究責任(分担)者もしくは研究協力者は症例報告書の変更・修正手順書に従い、データの変更もしくは修正を行う。

## **収集したデータの帰属**

本試験で得られた情報は国立循環器病研究センターに帰属し、研究責任者の許可なしに本情報へのアクセスを禁止する。

# **試料・情報の保存**

## **試料・情報の保管方法**

本試験で取得された試料・情報については下記の対応を遵守して管理する。

1. 試験情報（電子データ）は、研究責任者の責任の下、国立循環器病研究センターの情報セキュリティポリシーを遵守し管理する。具体的には、試験情報を保管する医局内個人デスクの PC及びストレージについては以下の措置を実施する。

・ウイルス対策ソフトの導入と最新化

・研究責任者が許可した研究者のみが利用可能とするための、IDとパスワードに

よるアクセス制限の実施

紙媒体の情報に関しては、研究責任者の責任の下、施錠可能である医局内個人デスクに保管する。

1. 試験試料である血液は、委託機関に送付するまで、研究責任者の責任の下、手術室10番の前の機材管理室の施錠されたフリーザー内で適切に管理する。

## **外部の機関との試料・情報の授受**

- - 試料・情報の授受の有無：□ 有　☑ 無

## **試料・情報の保存期間**

「国立循環器病研究センターにおける研究活動の不正行為への対応等に関する細則」に基づき、情報の保存期間は、論文発表後10年間とする。試料に関しては、残余は生じないため保管は行わない。

その後、紙媒体の情報はシュレッダー処理、溶解処分、電子データはデータ削除、データを保存した媒体の物理的破壊を行い、特定の個人を識別できないようにして廃棄する。

## **将来の研究への情報の利用**

本研究で取得した試料・情報は本研究のみに利用し、新たな研究への利用や提供は予定していない。ただし、もしその予定が生じた際は、研究計画書を作成し、倫理審査及び研究許可を受けて行なう。

# **品質管理と品質保証**

## **試験実施計画書の遵守・逸脱・変更**

研究責任(分担)者および研究協力者は、試験実施計画書に遵守して、本試験を遂行する。倫理審査委員会の承認を得ることなく、試験実施計画書から逸脱もしくは変更を行ってはいけない。逸脱した場合には、研究責任(分担)者もしくは研究協力者は、試験実施計画書から逸脱した行為を記録する。但し、医療上やむを得ないと判断された場合は、逸脱もしくは変更を行うことが出来る。

## **試験実施計画書の改訂**

　研究責任者は、改訂が必要と判断される情報を得た場合試験実施計画書を改訂する。改訂した試験実施計画書及びその改訂履歴（改訂内容とその理由）を作成する。改訂の際は、倫理審査委員会の承認及び研究機関の長の許可を得る。試験実施計画書の改訂中に被験者登録を中断する必要がある場合、研究責任者は研究分担者にその旨を連絡する。

## **モニタリング**

本試験では、研究責任者が指名するモニタリング担当者が、試験実施体制および実施状況についてモニタリングを実施する。詳細の手順は、別途定める手順書に記載する。

## **監査**

本試験では、国立循環器病研究センターの研究開発費のみを用いることから監査は行わない。

# **試験全体の終了もしくは中止**

## **試験全体の終了**

全解析が終了し、すべての論文投稿が完了した段階で、研究責任者は研究分担者・協力者等に試験全体の終了を通知する。研究責任者は、研究機関の長への報告を含めた終了手続きを実施する。

## **試験全体の中止・中断**

### **試験全体の中止・中断基準**

下記の基準に該当する場合に、研究責任者は試験全体を中止もしくは中断できる。試験を中止した時は、その中止の日から10日以内に、その旨を倫理審査委員会に通知する。

1. 重篤な有害事象の発生等により、被験者の安全性確保の観点より、本試験を継続して遂行することが困難であると研究責任者が判断した場合
2. 論文や学会発表等より本試験に関する安全性もしくは有効性に関する新たな情報が明らかとなり、試験全体の遂行が困難もしくは試験継続の意義がなくなったと研究責任者が判断した場合
3. 「人を対象とする生命科学・医学系研究に関する倫理指針」等の各種規範から著しく逸脱し、試験全体の遂行が困難であると研究責任者が判断した場合
4. 症例登録の遅れ等により本試験の完遂が困難であると研究責任者が判断した場合
5. 試験全体の中止が必要であると研究責任者が判断した場合

### **試験全体の中止・中断手順**

　試験の中止もしくは中断の基準に該当し、研究責任者が試験全体の中止もしくは中断が必要と判断した場合は、以下の手順で行う。

1. 研究責任者が試験全体の中止もしくは中断を決定した場合は、研究責任者は試験に携わるすべての者にその旨とその理由を速やかに報告する。
2. 研究責任者は、対象被験者に速やかにその旨を連絡し、然るべき対応を行う。加えて、研究責任者は、研究機関の長及び研究機関内の関連部署にその旨を報告し、当該研究機関の定められた手続きに従う。

# **試験の資金源及び利益相反**

## **試験の資金源**

本試験の資金は、国立循環器病研究センターから自己調達の形態で提供される。資金提供者である国立循環器病研究センターは、本試験の企画・立案、運営、発生するデータ及び結果の解析と解釈に関与することはない。

## **利益相反**

本試験は令和５年度循環器病研究開発費を資金源として実施され、企業等営利団体は関与しないため、開示すべき利益相反はない。

本試験に関する利益相反に関しては、医学研究に係る利益相反委員会に必要事項を申告し、その審査と承認を得るものとする。また、利益相反により被験者に不利益がもたらされないことを確認する。

# **健康被害の補償**

本試験に参加した結果として被験者に健康被害が生じた場合、研究機関はその治療に関する医療の提供等の適切かつ最善な対応を施す。本試験は通常診療で使用されている血液製剤を使用するため、副作用等の健康被害が出現した場合には通常診療として対応し、自己負担分の医療費、医療手当及び葬祭料や障害児補償金等の補償金は支払われない。また、医薬品副作用被害給付申請の対象となる場合がある。

本試験では、臨床研究保険に加入しない。

# **試験実施後における被験者への医療の提供に関する対応**

　試験終了後は、通常の保険診療での治療を継続する。

# **試験により得られた情報等の取り扱い**

本試験では、被験者に関わる健康に関する新たな結果等が得られないことを被験者等に説明する。

# **試験結果の公表と成果の帰属**

## **試験の登録**

　被験者登録開始前に、jRCTに本試験の概要を登録し、進捗状況や試験結果等の更新登録を行う。

## **試験成果の公表**

本試験の成果は、試験終了後に試験成果を公表する者等が論文や学会発表等にて公表する。試験成果を公表する者は、公表前に研究責任者や権利を有する者等と公表の必要性や公表内容を協議し、承認を得る。成果発表時には研究承認番号を記載する。

## **試験成果及びデータの帰属**

本試験により生じる知的財産権は、国立循環器病研究センター職務発明等規程に基づき、原則研究機関に帰属し、被験者やその家族には帰属しない。

# **試験の実施体制**

別紙：試験の実施体制参照

# **業務委託先の監督方法**

業務委託の有無：☑有　□無

1. 委託先機関名：SRL
2. 提供する項目：ヘパリン濃度測定
3. 委託業務内容：回収式自己血中のヘパリン濃度の測定

④　監督方法：委託業務の履行状況について、契約書等に従って行われているかどうかを適宜確認する。問題が認められたものについては必要な措置を講じるものとする。

# **参考文献**

1. 田中純子, 鹿野千治, 秋田智之, 杉山文, 栗栖あけみ. 献血の需要と供給の将来推計. 厚生労働科学研究費補助金（医薬品・医療機器等レギュラトリーサイエンス政策研究事業）令和２年度 研究報告書. 2020.

2. 的場聖明, 明石嘉浩, 香坂俊, 坂田泰史, 竹石恭知, 筒井裕之, et al. 循環器疾患診療実態調査（2020 年実施・公表） 報告書2020 October 1, 2025. Available from: https://www.j-circ.or.jp/jittai_chosa/media/jittai_chosa2019web_ver2_revise20241015.pdf.

3. Japan Society of Transfusion Medicine and Cell Therapy SoCSoTP. [Survey on the actual use of blood products, FY2019 report]2019. Available from: https://yuketsu.jstmct.or.jp/wp-content/uploads/2020/09/d888ba7e81de8e35f4fc1d1158f9a050.pdf.

4. Zacharias A, Habib RH. Factors predisposing to median sternotomy complications. Deep vs superficial infection. Chest. 1996;110(5):1173–8. doi: 10.1378/chest.110.5.1173. PubMed PMID: 8915216.

5. Leal-Noval SR, Marquez-Vácaro JA, García-Curiel A, Camacho-Laraña P, Rincón-Ferrari MD, Ordoñez-Fernández A, et al. Nosocomial pneumonia in patients undergoing heart surgery. Critical care medicine. 2000;28(4):935–40. doi: 10.1097/00003246-200004000-00004. PubMed PMID: 10809262.

6. Ranucci M, Pavesi M, Mazza E, Bertucci C, Frigiola A, Menicanti L, et al. Risk factors for renal dysfunction after coronary surgery: the role of cardiopulmonary bypass technique. Perfusion. 1994;9(5):319–26. doi: 10.1177/026765919400900503. PubMed PMID: 7833539.

7. Engoren MC, Habib RH, Zacharias A, Schwann TA, Riordan CJ, Durham SJ. Effect of blood transfusion on long-term survival after cardiac operation. The Annals of thoracic surgery. 2002;74(4):1180–6. doi: 10.1016/s0003-4975(02)03766-9. PubMed PMID: 12400765.

8. Dalrymple-Hay MJ, Pack L, Deakin CD, Shephard S, Ohri SK, Haw MP, et al. Autotransfusion of washed shed mediastinal fluid decreases the requirement for autologous blood transfusion following cardiac surgery: a prospective randomized trial. Eur J Cardiothorac Surg. 1999;15(6):830–4. doi: 10.1016/s1010-7940(99)00112-8. PubMed PMID: 10431866.

9. Wong JC, Torella F, Haynes SL, Dalrymple K, Mortimer AJ, McCollum CN. Autologous versus allogeneic transfusion in aortic surgery: a multicenter randomized clinical trial. Ann Surg. 2002;235(1):145–51. doi: 10.1097/00000658-200201000-00019. PubMed PMID: 11753054; PubMed Central PMCID: PMCPMC1422408.

10. Niranjan G, Asimakopoulos G, Karagounis A, Cockerill G, Thompson M, Chandrasekaran V. Effects of cell saver autologous blood transfusion on blood loss and homologous blood transfusion requirements in patients undergoing cardiac surgery on- versus off-cardiopulmonary bypass: a randomised trial. Eur J Cardiothorac Surg. 2006;30(2):271–7. Epub 20060707. doi: 10.1016/j.ejcts.2006.04.042. PubMed PMID: 16829083.

11. Shen S, Zhang J, Wang W, Zheng J, Xie Y. Impact of intra-operative cell salvage on blood coagulation in high-bleeding-risk patients undergoing cardiac surgery with cardiopulmonary bypass: a prospective randomized and controlled trial. Journal of Translational Medicine. 2016;14(1). doi: 10.1186/s12967-016-0986-6.

12. Lloyd TD, Geneen LJ, Bernhardt K, McClune W, Fernquest SJ, Brown T, et al. Cell salvage for minimising perioperative allogeneic blood transfusion in adults undergoing elective surgery. Cochrane Database Syst Rev. 2023;9(9):Cd001888. Epub 20230908. doi: 10.1002/14651858.CD001888.pub5. PubMed PMID: 37681564; PubMed Central PMCID: PMCPMC10486190.

13. Tomoko A, Koji K, Ikuko O, Kinari T, Seiko S, Saeko O, et al. INTRAOPERATIVE AUTOLOGOUS BLOOD COLLECTION AND AUTOTRANSFUSION FOR THE REDUCTION OF ALLOGENIC BLOOD TRANSFUSION CARDIOVASCULAR SURGERY. Japanese Journal of Transfusion and Cell Therapy. 2017;63(5):674–82.

14. Pagano D, Milojevic M, Meesters MI, Benedetto U, Bolliger D, von Heymann C, et al. 2017 EACTS/EACTA Guidelines on patient blood management for adult cardiac surgery. Eur J Cardiothorac Surg. 2018;53(1):79–111. doi: 10.1093/ejcts/ezx325. PubMed PMID: 29029100.

15. Tibi P, McClure RS, Huang J, Baker RA, Fitzgerald D, Mazer CD, et al. STS/SCA/AmSECT/SABM Update to the Clinical Practice Guidelines on Patient Blood Management. The Annals of thoracic surgery. 2021;112(3):981–1004. Epub 20210630. doi: 10.1016/j.athoracsur.2021.03.033. PubMed PMID: 34217505.

16. Horan TC, Andrus M, Dudeck MA. CDC/NHSN surveillance definition of health care-associated infection and criteria for specific types of infections in the acute care setting. Am J Infect Control. 2008;36(5):309–32. doi: 10.1016/j.ajic.2008.03.002. PubMed PMID: 18538699.

17. 奥田誠, 池本純子, 石丸健, 内川誠, 梶原道子, 北澤淳一, et al. 赤血球型検査（赤血球系検査）ガイドライン（改訂 4 版）. 日本輸血細胞治療学会誌. 2022;68:539–56.
