# Supplementary material for: Intraoperative cell-salvaged versus allogeneic red blood cell transfusions in high-bleeding-risk cardiovascular surgery: Protocol for a single-center, randomized, parallel-group, noninferiority trial
Source: PLoS One. 2025 Oct 24;20(10):e0334397. doi: 10.1371/journal.pone.0334397 (PMC12551814; doi:10.1371/journal.pone.0334397)
Supplement: Attachment — Submitted filename: Response to Reviewers_ver6.docx [file pone.0334397.s004.docx]

Manuscript Number: PONE-D-25-26936

Intraoperative cell-salvaged versus allogeneic red blood cell transfusions in high-bleeding-risk cardiovascular surgery: Protocol for a single-center, randomized, parallel-group, non-inferiority trial

August 22, 2025

Dear Dr Baysal,

Attached is a revised version of our manuscript titled “**Intraoperative cell-salvaged versus allogeneic red blood cell transfusions in high-bleeding-risk cardiovascular surgery: Protocol for a single-center, randomized, parallel-group, non-inferiority trial**,” which we would like to resubmit for publication in *PLOS ONE*.

Your comments were highly insightful and enabled us to greatly improve the quality of our manuscript. We present our point-by-point responses to the comments on the following pages.

In accordance with your suggestions, we have revised some text for better clarity. We hope that our revisions and accompanying responses will be sufficient to make our manuscript suitable for publication in *PLOS ONE*.

We look forward to hearing from you at your earliest convenience.

Yours sincerely,

Akito Tsukinaga

Email: akito.tsukinaga30303@gmail.com

**Responses to the Academic Editor**

**Comment:** One of the main concerns with the manuscript is how to overcome bias and missing data. The authors should mitigate, and account for potential biases and missing data in their study to ensure robust, reliable, and generalizable findings.

**Response:**

**1. Potential Bias:**

We understand the Academic Editor’s concern regarding potential bias in the study, particularly related to intraoperative transfusion management. Because the attending anesthesiologist, who is responsible for transfusion decisions, cannot be blinded, there is a risk of performance bias in the amount of RBCs administered, even when hemoglobin thresholds are predefined. Recognizing this limitation, we deliberately avoided using RBC transfusion volume as the primary outcome. Instead, we selected postoperative chest tube blood loss within 12 hours from the end of surgery as the primary endpoint, as this is an objective and quantifiable measure that is less susceptible to clinician discretion. While RBC transfusion volume remains a secondary outcome and will be analyzed accordingly, we do not intend to draw definitive conclusions based solely on this measure. We believe that this outcome selection minimizes the risk of performance bias and strengthens the internal validity of the study.
We have added this rationale to the revised manuscript (Page 32, Lines 548–552 in clean version).

**2. Missing Data:**
We recognize that missing data may introduce bias and reduce the reliability of the findings. Missing data in the primary and secondary outcomes are expected to be rare because these data are collected as part of routine clinical practice. Therefore, we will use complete case analysis as the primary approach. However, if the rate of missing data exceeds 5%, we will evaluate the mechanism of missingness and perform sensitivity analyses using appropriate imputation methods, such as multiple imputation. This approach aligns with best practices and enhances the robustness of our findings. The revised plan is now clearly stated in the manuscript (Page 27, Lines 458–462 in clean version).

We believe these considerations will ensure the internal validity and reliability of our findings. Relevant revisions have been incorporated into the manuscript.

**Responses to Reviewer #1**
**Point 1:**

Title: I don't believe "safety" correctly describes the final aim of the paper. As the authors state, this is a non-inferiority trial with blood loss as primary endpoint. The safety outcomes are mentioned within the manuscript, after the primary and the secondary outcomes, and only the "prevalence of any infection" has to do with actual safety. Indeed, no other Severe Adverse Events that might be evaluated as safety among the two groups are described. The title should be better formulated to reflect this aspect.

**Response:**

We thank the reviewer for this valuable comment. We agree that the term “safety” in the title may not accurately reflect the study’s primary aim, which is to evaluate the noninferiority of salvaged red blood cell (RBC) transfusion compared with allogeneic RBC transfusion in terms of postoperative blood loss. In response, we revised not only the title but also the relevant wording in the abstract to ensure consistency and better reflect the objective of evaluating a clinically relevant outcome, rather than emphasizing safety (Page 1, Lines 1–3 and Page 3, Lines 35–39 in clean version).

**Point 2:**

Exclusion criteria: Please define "extremely high risk of bleeding".

**Response:**

We appreciate this being pointed out. We agree that the term “extremely high risk of bleeding” requires a clear operational definition to enhance reproducibility and transparency. On the basis of our institutional experience and the clinical records of previously excluded cases, we define “extremely high risk of bleeding” as either a preoperative platelet count of < 100 × 10⁹/L, or ≥ 2 prior cardiovascular surgeries via median sternotomy.

We have added this definition in the exclusion criteria in the revised manuscript (Page 8, Lines 116–117 in clean version). We believe this clarification improves reproducibility and transparency. Because we have provided a clear definition of “extremely high risk of bleeding,” the third limitation regarding the subjectivity of this criterion has been removed from the revised manuscript (Page 32, Line 557 in clean version).

**Point 3:**
Please add a paragraph about the management of anemic patients that need to be transfused during CPB (are they transfused preoperatively or perioperatively? are they excluded or maintained in the study).

**Response:**
We thank the reviewer for this important question. We have clarified the perioperative management of anemic patients in the “Intervention – concomitant care” section (Page 12, Lines 195–197 in clean version). Treatment for preoperative anemia, including RBC transfusion, is not defined by the study protocol; rather, the decision regarding this therapy is left to the discretion of the attending physician based on clinical judgment, such as symptoms of anemia or underlying comorbidities. Because this decision will be made before final registration and randomization, it is unlikely to introduce bias into the trial.

If an anemic patient proceeds to surgery without a preoperative transfusion, hemodilution during CPB may reduce hemoglobin levels below the predefined threshold of 8 g/dL, prompting intraoperative transfusion and qualifying the patient for final registration. We acknowledge that this design may allow enrollment of patients with low actual intraoperative bleeding risk—particularly those with preoperative anemia or small body size—if they require transfusion primarily because of hemodilution rather than bleeding. Such patients were not excluded because their exclusion could have markedly reduced the number of eligible participants and compromised study feasibility. To address this limitation, we plan to perform subgroup analyses on the basis of postoperative bleeding volume after CPB weaning, which may serve as an indirect indicator of actual transfusion requirements. This information has been added to the “Limitations” section of the revised manuscript (Page 32–33, Line 557–565 in clean version).

**Point 4:**
Is there a bleeding management algorithm including point-of-care testing in the Institution?

**Response:**
We thank the reviewer for this important question. Although our institution has access to point-of-care coagulation testing devices, such as Teg® 6s (Haemonetics, Boston, MA, USA), Quantra® Hemostasis Analyzer (HemoSonics, Charlottesville, VA, USA), and Fibcare® (Atom Medical, Kawasaki, Japan), there is no standardized bleeding management algorithm based on these platforms. Instead, clinical decisions during surgery are made at the discretion of the attending anesthesiologist. To minimize inter-physician variability and ensure consistent perioperative management during the study, we have clarified and standardized our transfusion practices by establishing a protocol-based strategy (Page 13, Line 221 and Page 14, Lines 223–236 in clean version).

**Point 5:**
How FFP or Platelet Concentrate transfusion managed if it is required immediately after CPB weaning (because anticipated due to patient's preoperative paramenters or to uncontrolled bleeding after CPB weaning).

**Response:**

We thank the reviewer for this valuable question. We have provided a detailed description of the management of FFP and platelet concentrate transfusions in the revised manuscript (Page 13, Line 221 and Page 14, Lines 223–236 in clean version).
Additionally, to guide transfusion management and assess coagulation status, we will perform not only standard laboratory tests but also point-of-care testing, such as blood gas analysis and ACT, with viscoelastic testing at specific perioperative time points. These details are described in the newly added “Intervention – Laboratory and Viscoelastic Testing” section (Page 15–17, Line 249–282 in clean version).

**Point 6:**
Blood viscosity testing is mentioned among the Safety outcomes. Please give more technical details of how and when this testing is performed, the parameters that will be considered abnormal/at risk, and how this affects safety of the patient.

**Response:**

We thank the reviewer for making this insightful comment. Based on the feedback, we have revised the protocol to reclassify viscoelastic testing from a safety outcome to an exploratory outcome (Page 19, Line 316–319 in clean version). This change was made because the primary role of viscoelastic testing in this study is to provide supportive, hypothesis-generating information rather than to directly assess patient safety. If noninferiority of the primary endpoint (postoperative chest tube blood loss) is not demonstrated, analysis of these exploratory parameters may help identify potential mechanisms, such as residual heparin effect (elevated clot time ratio), thrombocytopenia or platelet dysfunction (reduced platelet contribution to clot stiffness), or hypofibrinogenemia/fibrinogen dysfunction (reduced fibrinogen contribution to clot stiffness).

In response, we have added related technical details in a newly created subsection titled “Intervention – Laboratory and Viscoelastic Testing” (Page 16–17, Line 269–282 in clean version) in the revised manuscript. This section describes how and when the testing will be performed, which parameters will be considered abnormal or indicative of risk, and how these findings will relate to patient safety.

**Responses to Reviewer #2**

**Point 1:**

Please provide justification for the non-inferiority margin of 200 ml.

**Response:**

We thank the reviewer for this comment. The noninferiority margin of 200 mL was determined on the basis of our institutional data and previous studies. According to our preliminary data, patients who met the eligibility criteria for this trial and received only allogeneic RBC transfusions had a mean postoperative chest tube blood loss of 674 mL within 12 hours from the end of surgery. This corresponds to Class 1 bleeding (601–800 mL) as defined by the Universal Definition of Perioperative Bleeding (UDPB) proposed by Mehta et al. (Ann Thorac Surg. 2009;87:148–156). The UDPB categorizes bleeding severity in approximately 200-mL increments: the threshold between Class 0 (insignificant) and Class 1 (mild) is 600 mL; Class 2 (moderate) begins at 801 mL, and Class 3 (severe) begins at 1001 mL. On the basis of these clinically meaningful thresholds, we conservatively selected 200 mL as the noninferiority margin.
We have added this rationale to the main manuscript (Page 21, lines 349–355 in clean version).

**Point 2:**

Authors plan to exclude the patients with extremely high bleeding risk. Please define or how to identify “extremely high risk” here.
**Response:**

We thank the reviewer for this helpful comment. We agree that the term “extremely high risk of bleeding” requires a clear operational definition to enhance reproducibility and transparency. On the basis of our institutional experience and clinical records of previously excluded cases, we define “extremely high risk of bleeding” as either a preoperative platelet count of < 100 × 10⁹/L, or ≥ 2 prior cardiovascular surgeries via median sternotomy. We have added this definition to the exclusion criteria in the revised manuscript (Page 8, Lines 116–117 in clean version). We believe this clarification improves reproducibility and transparency. Because we have now provided a clear definition of “extremely high risk of bleeding,” the third limitation regarding the subjectivity of this criterion has been removed from the revised manuscript (Page 32, Line 557 in clean version).

**Point 3:**

It would be great to lay out data access process.
**Response:**

We appreciate this valuable suggestion. In response, we have revised the manuscript to clearly describe the data access process in accordance with the SPIRIT 2013 guidelines. Specifically, we have updated the “Access to data” section (Page 30, Line 509–514 in clean version) to clarify data access during the trial. Furthermore, we have revised the “Data Availability Statement” section (Page 34, Lines 586–589 in clean version) to specify that, upon completion of the trial, the deidentified dataset will be deposited in a publicly accessible repository (i.e., Dryad) to facilitate verification, replication, and secondary analyses by other researchers.

**Point 4:**

Please comment on how to deal with missing data.

**Response:**

We appreciate the comment regarding missing data. We recognize that missing data may introduce bias and reduce the reliability of the findings. Missing data in the primary and secondary outcomes are expected to be rare because these data are collected as part of routine clinical practice. Therefore, we will use complete case analysis as the primary approach. However, if the rate of missing data exceeds 5%, we will evaluate the mechanism of missingness and perform sensitivity analyses using appropriate imputation methods, such as multiple imputation. This approach aligns with best practices and enhances the robustness of our findings. The revised plan is now clearly stated in the manuscript (Page 27, Lines 458–462 in clean version).

**Responses to Reviewer #3**

**Point:** In the present manuscript, Tsukinaga et al describe a study protocol designed to compare the respective values of autologous versus allogeneic transfusion in the setting of high risk cardiac surgery. The question is of interest and the results might be of interest to the reader. However the protocol itself has structural flaws, especially the consideration of the primary endpoint wich is irellevant to the question, and the manuscript presents no data/result. Therefore it does not represent a work worthy of publication in its current state.

**Response:**

We appreciate the thoughtful review and feedback. We respectfully disagree with the assertion that our protocol has structural flaws or that the primary endpoint is irrelevant to the study question.

Our primary objective is to assess whether intraoperative transfusion of cell-salvaged red blood cells (RBCs) is noninferior to allogeneic RBCs in terms of perioperative bleeding risk. As such, we selected postoperative chest tube blood loss within 12 hours from the end of surgery as the primary endpoint, which is a direct and objective measure of early postoperative bleeding. This endpoint is clinically relevant and has been used in previous transfusion-related studies to evaluate hemostatic outcomes (e.g., A. Laine et al., J Cardiothorac Vasc Anesth 2018;32:131-139; A.W. Bracey et al., Transfusion 1999;39:1070–1077).

We also understand that protocol manuscripts do not contain results. Our manuscript follows the SPIRIT (Standard Protocol Items: Recommendations for Interventional Trials) 2013 guidelines and is intended to present a detailed and transparent plan for a randomized controlled trial before the study is completed.

We hope that with these clarifications, the scientific rationale and structure of the protocol will be viewed more favorably.

**Responses to Reviewer #4**

**Point 1:** I thought this was a very clear, well written protocol. My only concern is with regard to question 4. The authors state that the data will only be available to the principal investigator and statistician. They need to explain how the anonymized data will be publicly available.

**Response:**

We appreciate the kind comments and the constructive suggestion. In response, we have revised the manuscript to clearly describe the data access process in accordance with the SPIRIT 2013 guidelines. Specifically, we have updated the “Access to data” section (Page 30, Line 509–514 in clean version). Additionally, we have revised the “Data Availability Statement” section (Page 34, Lines 586–589 in clean version) to state that deidentified participant-level data will be deposited in the Dryad digital repository upon completion of the trial, ensuring public access for secondary analysis by other researchers.

**Point 2:**

A secondary minor comment: pay attention to the use of capital letters in journal titles. All significant words should be capitalized.

**Response:**
We appreciate this comment. In response, we have carefully reviewed and revised all references to ensure that the correct parts of the journal titles are properly capitalized, in accordance with the journal’s style guidelines.

**Responses to Reviewer #5**
**Point 1:**

Defining ‘High risk bleeding’ and ‘extremely high bleeding risk’ in the inclusion and exclusion criteria of the study population

**Response:**

We thank the reviewer for this important comment. In our original protocol, “high bleeding risk” was operationally defined by the inclusion of specific cardiac surgical procedures that are associated with an increased risk of perioperative bleeding (e.g., complex aortic surgery, redo cardiac surgery). This procedure-based classification is based on the study by Shen et al. (J Transl Med. 2016;14:1), which categorized patients as having a high bleeding risk on the basis of surgical type.

To further clarify the exclusion criteria, we have provided an operational definition of “extremely high bleeding risk” using specific clinical characteristics, as follows: “*we define “extremely high risk of bleeding” as either a preoperative platelet count of < 100 × 10⁹/L, or ≥ 2 prior cardiovascular surgeries via median sternotomy*” (Page 8, Lines 116–117 in clean version). By clearly distinguishing this subgroup, we believe the conceptual boundary between “high” and “extremely high” bleeding risk is now well described in the manuscript, thereby enhancing the transparency and reproducibility of our study design.

We hope this two-tiered classification adequately addresses this concern.

**Point 2:**

The anaesthesiologist, who is aware of the allocation, also manages transfusions. This can result in RBC usage bias.

**Response:**

We appreciate this important comment. We agree that the unblinded status of the attending anesthesiologist, who is responsible for intraoperative transfusion management, may introduce performance bias, particularly regarding RBC usage. However, masking the treatment allocation from the anesthesiologist was considered infeasible because of the nature of the intervention.

To mitigate this potential bias, we implemented two strategies:

1. **Standardization of transfusion protocols:**
   To minimize variation in perioperative transfusion practices, we established detailed, protocol-defined transfusion criteria not only for RBCs, but also for other interventions that may influence postoperative bleeding (e.g., transfusion of fresh frozen plasma, platelet concentrates, antifibrinolytics such as tranexamic acid). These efforts were designed to ensure consistency in transfusion management across both study groups.
2. **Objective selection of the primary outcome:**
   Recognizing the susceptibility of transfusion volume to performance bias, we deliberately selected an objective and quantifiable primary outcome, postoperative chest tube blood loss within 12 hours from the end of surgery, which is less likely to be influenced by clinician judgment. Transfusion volume is treated as a secondary outcome for this reason.

Despite these precautions, we acknowledge that the risk of performance bias cannot be eliminated entirely. We consider this one of the most important limitations of our study.
These considerations are addressed in the revised manuscript. The first point was discussed in the original limitations section, and the second has been added for completeness (Page 32, Lines 548–552 in clean version).

**Point 3:**

Rationale for taking 200 ml as cut off between the salvaged and allogeneic transfused group.
**Response:**

We appreciate this valuable comment. The noninferiority margin of 200 mL was determined using our institutional data and the findings in previous studies. According to our preliminary data, patients who met the eligibility criteria for this trial and received only allogeneic RBC transfusions had a mean postoperative chest tube blood loss of 674 mL within 12 hours from the end of surgery. This corresponds to Class 1 bleeding (601–800 mL) as defined by the Universal Definition of Perioperative Bleeding (UDPB) proposed by Dyke et al. (The Journal of Thoracic and Cardiovascular Surgery 2014;147:1458). The UDPB categorizes bleeding severity in approximately 200-mL increments: the threshold between Class 0 (insignificant) and Class 1 (mild) is 600 mL; Class 2 (moderate) begins at 801 mL, and Class 3 (severe) begins at 1001 mL. On the basis of these clinically meaningful thresholds, we conservatively selected 200 mL as the noninferiority margin.
We have added this rationale to the main manuscript (Page 21, lines 349–355 in clean version).

**Point 4:**

The protocol suggests patients with major protocol violations will be excluded from PP analysis, but doesn’t explain how many deviations are expected or how these will be minimized.
**Response:**

As described in the revised “Statistical Methods” section, the per-protocol analysis set is defined as a subset of the modified intention-to-treat population, from which patients with major protocol violations—such as violations of eligibility criteria or improper administration of the study intervention—will be excluded. We acknowledge that the original wording in the manuscript may have been misleading, and we have revised the text to improve clarity (Page 25, Line 424–427 in clean version).

Major violations related to the inclusion/exclusion criteria or the protocol for salvaged/allogeneic RBC transfusion are not anticipated. However, we estimate that violations involving pre-, concomitant, or post-treatment may occur in approximately 5% of cases. To minimize such violations, we will provide a detailed explanation of the study procedures to all relevant staff prior to each surgical case. This approach ensures consistent understanding and protocol adherence. Additionally, protocol compliance will be monitored through regular investigator meetings and ongoing review of accumulated study data, enabling early detection and correction of any violations. These details are described in the newly added “Intervention – adherence” section (Page 17–18, Lines 283–294 in clean version).

**Point 5:**

Rationale for choosing liberal transfusion triggers while recent studies advocate restrictive transfusion protocols.
**Response:**

This study targets patients undergoing cardiac surgery with a high risk of bleeding. Perioperative anemia is associated with adverse outcomes, and in such high-risk populations, a restrictive transfusion strategy may increase the risk of unintended prolonged anemia. Therefore, a liberal transfusion threshold was adopted to prioritize patient safety by reducing the risk of anemia-related complications. This approach allows for a more physiologically stable patient condition in which to evaluate the noninferiority of salvaged blood in terms of postoperative bleeding. We have revised the “Interventions” section accordingly (Page 9, Lines 145–149 in clean version).

**Point 6:**

Volume of transfusion (salvaged blood vs allogeneic blood) in both the study arm may differ and might affect the outcome.
**Response:**

We appreciate this thoughtful comment regarding differences in transfusion volume between the two groups and the potential impact on outcomes.

Differences in RBC transfusion volume alone may affect bleeding outcomes. As described in the Introduction section, the hematocrit of salvaged RBC solutions is slightly lower than or comparable to that of packed allogeneic RBCs. According to the manufacturer’s technical manual (Haemonetics Cell Saver Elite+ Operator’s Manual, 2024), the average hematocrit of salvaged RBCs is approximately 56% ± 0.3%, which corresponds to a hemoglobin concentration of approximately 18.7 g/dL [8]. In contrast, the reported hemoglobin content of packed allogeneic RBCs was approximately 21 g/dL (SD 2.9) in a prospective study by Jain et al. (2022) [9] and approximately 19.1 g/dL (SD 0.7) in Japanese donors, as described in the Japanese Red Cross Society guidelines (2009) [10]. These differences suggest that patients in the salvage group may be more prone to dilutional coagulopathy. However, because the differences in hemoglobin concentration between the two types of RBC preparations are small, we believe that the clinical impact is likely to be limited. Additionally, the potential bias from hemodilution in the salvage group would likely increase postoperative bleeding, thereby reducing the likelihood of demonstrating noninferiority. Therefore, we believe there is a low risk of falsely confirming our hypothesis because of this bias. That said, we agree that this point represents an important limitation of the study, and we have accordingly added it to the "Limitations" section of the manuscript (Page 33, Line 565–571 in clean version).

**Point 7:**

Salvaged blood has a higher risk of hemolysis and infection. Specifying reporting and monitoring of adverse event method, agency and an audit strategy would promote transparency.

**Response:**

We appreciate this valuable comment regarding the need to clarify the methods for reporting and monitoring adverse events, particularly in relation to the potential risks of hemolysis and infection associated with salvaged RBC transfusion.

To address all relevant concerns, we have revised the manuscript to include the following:

1. **Safety Outcomes:**
   We have added the following hemolysis-related indicators: gross hematuria upon ICU admission and postoperative acute kidney injury defined in accordance with the KDIGO criteria to the revised “Safety Outcomes” subsection (Page 18–19, Line 310–314 in clean version).
2. **Monitoring and Reporting of Adverse Events:**
   We have clarified that all adverse events, including serious adverse events, will be promptly documented by the investigators in the case report forms and reviewed for causality and expectedness. Regular monitoring will be performed by designated staff to confirm protocol compliance and assess patient safety. These staff members will be independent of the sponsor and will have no competing interests. Although external auditing is not planned, internal audits will be performed periodically.

These additions are reflected in the revised **“Safety Considerations”** subsection (Page 28 , Line 474–485 in clean version). Additionally, detailed definitions and procedures for evaluating and reporting adverse events and serious adverse events are provided in Supplementary Files S2 (English version) and S3 (Japanese version), as mentioned in the revised “Safety Considerations” section.

We hope these modifications enhance the clarity and transparency of our study protocol regarding the assessment and oversight of adverse events related to salvaged blood transfusion.
