# Supplementary material for: Intraoperative cell-salvaged versus allogeneic red blood cell transfusions in high-bleeding-risk cardiovascular surgery: Protocol for a single-center, randomized, parallel-group, noninferiority trial
Source: PLoS One. 2025 Oct 24;20(10):e0334397. doi: 10.1371/journal.pone.0334397 (PMC12551814; doi:10.1371/journal.pone.0334397)
Supplement: Attachment — Submitted filename: Response to Reviewers.docx [file pone.0334397.s005.docx]

Manuscript Number: PONE-D-25-26936

Intraoperative cell-salvaged versus allogeneic red blood cell transfusions in high-bleeding-risk cardiovascular surgery: Protocol for a single-center, randomized, parallel-group, non-inferiority trial

September 23, 2025

Dear Dr Baysal,

Attached is a revised version of our manuscript titled “**Intraoperative cell-salvaged versus allogeneic red blood cell transfusions in high-bleeding-risk cardiovascular surgery: Protocol for a single-center, randomized, parallel-group, non-inferiority trial**,” which we would like to resubmit for publication in *PLOS ONE*.

Your comments were highly insightful and enabled us to greatly improve the quality of our manuscript. We present our point-by-point responses to the comments on the following pages.

In accordance with your suggestions, we have revised some text for better clarity. We hope that our revisions and accompanying responses will be sufficient to make our manuscript suitable for publication in *PLOS ONE*.

We look forward to hearing from you at your earliest convenience.

Yours sincerely,

Akito Tsukinaga

Email: akito.tsukinaga30303@gmail.com

**Responses to Reviewer #2**

**Comment:** Thanks for appropriately addressing all the comments. This reviewer has no further concerns on this revised manuscript.

**Response:**

We sincerely thank the reviewer for their positive evaluation and are grateful for their supportive comments.

**Responses to Reviewer #3**

**Point 1:** While the authors have addressed most of the reviewers’ comments, some of their responses introduce additional concerns. For example, the definition of “extremely high risk of bleeding” combines a reasonable criterion (two or more prior surgeries) with another that is less appropriate (platelet count below 100). The latter does not in itself confer an extremely high bleeding risk and can be readily managed with preoperative platelet transfusion.

**Response:** We appreciate the reviewer’s insightful comment. In response, we have removed the platelet count <100 × 10⁹/L criterion and now define “extremely high risk of bleeding” solely as patients with two or more prior sternotomies. The initial rationale for including the platelet count threshold was to exclude patients with coagulopathy, such as chronic disseminated intravascular coagulation (DIC) associated with aortic aneurysm, since these patients may still exhibit a bleeding tendency intraoperatively even if preoperative platelet transfusion normalizes the platelet count. However, as the reviewer correctly noted, not all patients with platelet counts below 100 × 10⁹/L necessarily present with coagulopathy. At the same time, it is difficult to establish a clear and objective definition to specifically exclude cases of coagulopathy associated with aortic aneurysm. Therefore, in the revised manuscript, we have clarified that for patients considered to be at extremely high risk of bleeding, the final decision on exclusion will be made through discussion between the surgeon and the anesthesiologist.

We have revised the *Exclusion criteria* section accordingly, which now reads as follows:
*“Extremely high risk of bleeding, defined as patients with two or more prior cardiovascular surgeries via median sternotomy. For other cases in which the bleeding risk is considered to be extremely high, the final decision on exclusion will be made through discussion between the surgeon and the anesthesiologist.”* (Page 8, Lines 116–119 in clean version)
**Point 2:** More generally, the authors—likely surgeons and anesthesiologists—would benefit from broadening the interdisciplinary basis of their research and involving colleagues such as transfusion medicine specialists and hematologists in order to refine their study protocol and ensure its relevance to clinical practice.

**Response:** We thank the reviewer for this thoughtful suggestion. We would like to clarify that the first author (AT) and co-author (KY) are anesthesiologists with expertise in transfusion medicine, as they are affiliated with the Department of Transfusion. We have drawn upon this expertise in planning the present study protocol.

**Responses to Reviewer #**4:

**Comment:** Thank you for the opportunity to again review this protocol. The edits made by the authors have clarified the questions asked after the initial review. This well-designed study will be of great interest to anyone who is interested in the optimal use of perioperative blood salvage.

**Response:** We sincerely thank the reviewer for the positive evaluation and encouraging comments regarding our study.

**Point 1:** Just a point of clarification, on page 10, lines 161 -163: When you say that you will set up the cell saver, will that be done for all cases, whether or not it will be used? For the sake of preserving the blinding, it would need to be set up for even the control cases and a sham procedure (i.e. running of the instrument) should be done when salvaged blood will not be processed or transfused so that the blinded participants in the room will not know if it is actually being used or not.

**Response:** We thank the reviewer for this valuable suggestion. We agree that, in order to preserve blinding, the Cell Saver should be prepared and operated in both the intervention and control groups. Accordingly, we have revised the manuscript to clarify this point. The revised text now reads:

1. “In accordance with the instruction manual, we will prepare the Cell Saver Elite+ (Haemonetics, Boston, MA, USA) and install the cell salvage disposables in all cases, regardless of allocation to the intervention or control group, to perform the intraoperative cell salvage procedure.” (Page 10, Lines 158–161 in clean version)
2. “In the control group, the same collection, washing, and storage in the reinfusion bag will be performed as in the intervention group, in order to preserve blinding.” (Page 11–12, Lines 183–184 in clean version)

**Point 2:** Another point of clarification, on page 18, line 309, the first secondary outcome: This might be more of an exploratory outcome. For the study group, will you record how much of the RBC volume transfused is from cell salvage vs. banked RBCs? It would be interesting to see if there is a tendency to see more bleeding in cases in which a larger proportion of RBCs transfused comes from salvaged blood

**Response:** We thank the reviewer for this valuable comment. We selected intraoperative and early postoperative RBC transfusion volume as a secondary outcome because, if our hypothesis holds true that the use of salvaged RBCs does not increase bleeding compared with allogeneic transfusion, then the need for allogeneic RBCs should be reduced in the intervention group. Thus, this outcome is directly linked to verifying the clinical relevance of our hypothesis.

As suggested, we will also record, for the intervention group, the transfused RBC volume separately for cell-salvaged and allogeneic sources. Furthermore, we agree that it is of interest to examine whether cases receiving a larger proportion of salvaged RBCs show different bleeding tendencies. To address this point, we have already planned a subgroup analysis of the primary outcome according to the total volume of intraoperative salvaged and allogeneic RBC transfusions after weaning from CPB (≥ 800 mL vs. < 800 mL). We believe this will provide exploratory insights into the reviewer’s concern.

To clarify this in the manuscript, we have revised the *Data collection and management* section as follows:

*“Other data, including baseline characteristics and all secondary outcomes, will be collected from the electronic database. Among these, the total RBC transfusion volume will be recorded for all patients, and in the intervention group, the transfused RBC volume will additionally be documented separately as cell-salvaged and allogeneic sources. All data will be entered into the respective CRF by unblinded researchers.”* (Page 24–25, Lines 414–418 in clean version)

**Responses to Reviewer #**5

Comment: All suggestions have been considered. I believe the manuscript has improved in clarity and scientific value.

Response: We sincerely thank the reviewer for their positive evaluation and are grateful for their supportive comments.
